# Digital health interventions for promoting adults lifestyle behaviors: who is being left behind? An evidence synthesis of social inequality

**DOI:** 10.1186/s12966-026-01874-4

**Published:** 2026-02-06

**Authors:** Jiajun Jiang, Qiying Zhong, Zhihua Yin, Qingyuan Zhou, Yanfang Wang, Zijun Yan

**Affiliations:** East China Normal University, College of Physical Education and Health, Shanghai, 200241 China

**Keywords:** Digital age, Digital health interventions, Lifestyle behaviors, Social inequality, Umbrella review

## Abstract

**Background:**

Digital health interventions have gained increasing prominence worldwide and demonstrate substantial potential for promoting healthy lifestyle behaviors. However, accumulating evidence suggests that not all population groups benefit equally from these interventions, raising concerns about persistent social inequalities in access, engagement, and outcomes.

**Methods:**

This study conducted an umbrella review to systematically synthesize evidence from review-level studies that examined social inequality indicators in digital health interventions targeting lifestyle behaviors among adults. Comprehensive searches were performed across seven electronic databases, identifying 41 eligible reviews published between January 2000 and June 2025. Data were extracted on targeted behavioral domains, intervention outcomes, and reported social inequality indicators.

**Results:**

The included reviews primarily focused on interventions targeting physical activity and diet, followed by sedentary behavior and sleep, with behavioral outcomes serving as the main evaluation metrics. Among social inequality indicators, age, gender, and place of residence were most frequently reported. In contrast, indicators such as income, race/ethnicity, socioeconomic status, education, digital health literacy, and employment were substantially underrepresented. This uneven distribution indicates significant gaps in the current evidence base and suggests that the differential effects of digital health interventions across social groups may be underestimated or overlooked.

**Conclusions:**

Current review-level evidence on digital health interventions insufficiently captures the full spectrum of social inequalities shaping intervention access, engagement, and benefits. To support more equitable and inclusive health promotion strategies, future research should systematically incorporate a broader range of social inequality indicators and conduct in-depth analyses of the mechanisms underlying unequal intervention effects across diverse demographic and socioeconomic groups.

**Supplementary Information:**

The online version contains supplementary material available at 10.1186/s12966-026-01874-4.

## Introduction

In recent decades, lifestyle-related risk factors, including insufficient physical activity, prolonged sedentary behavior, unhealthy dietary patterns, and sleep disorders, have continued to rise globally. Accumulating evidence indicates that insufficient physical activity and prolonged sedentary time substantially increase the risks of obesity, type 2 diabetes, cardiovascular disease, selected cancers, and premature mortality [1–3]. Unhealthy dietary patterns, which involve high intakes of sugar and fat alongside insufficient consumption of fruits and vegetables, contribute substantially to metabolic syndrome, obesity, and chronic systemic inflammation [4, 5]. Concurrently, insufficient sleep duration or poor sleep quality has been consistently associated with a range of adverse health outcomes, including obesity, depression, cardiovascular disease, and cognitive decline [6, 7]. Collectively, these adverse lifestyle behaviors not only pose sustained threats to individual health but also contribute to the growing prevalence of chronic diseases, thereby increasing demand for healthcare services, long-term care expenditures, and health resource utilization. As a result, these behaviors constitute a major source of disease burden for global public health systems [8, 9]. Given that population-level lifestyle interventions are frequently constrained by limitations related to resources, costs, accessibility, and long-term sustainability, there is a pressing need to explore alternative, scalable approaches to health promotion. In this context, digital health interventions have emerged as a promising pathway for addressing these challenges. By leveraging technologies such as mobile health applications, wearable devices, and remote consultation platforms, digital health interventions offer advantages in terms of cost-effectiveness, operational efficiency, and scalability [10, 11]. Moreover, these interventions enable a higher degree of personalization, thereby demonstrating substantial potential for effective health promotion [12].

Numerous evidence suggests that digital health interventions can produce favorable effects in improving lifestyle behaviors [13–17]. However, the effectiveness of these interventions is not uniformly distributed across different population groups [18]. Variations in individual-level factors, including digital health literacy, access to technology, device availability, and affordability, may place certain population groups at a disadvantage in accessing and utilizing digital health resources [19]. Even in countries with high levels of broadband and smartphone penetration, substantial gaps persist in digital skills and effective technological access among older adults, low-income populations, rural residents, and individuals with lower education [20, 21]. These disparities not only undermine the accessibility and sustainability of digital health interventions but may also lead to disparate outcomes in behavior change and health improvement, thereby exacerbating existing health inequality [22, 23]. From a broader perspective, health-related social inequality indicators are not merely technological issues but are deeply embedded within broader socioeconomic structures and policy environments. Extensive research demonstrates that income, education, employment, place of residence, and social protection coverage consistently shape individuals’ exposure to health risks, access to health services, and capacity to benefit from health interventions [24–28]. Reports from the WHO and the OECD indicate that although health equality goals have been widely acknowledged at the policy level, actual health inequality has not substantially narrowed in many regions and has even widened among certain populations [29, 30].

Across regions, the institutional contexts shaping social inequality indicators and corresponding policy responses vary substantially. In welfare states, particularly in Northern Europe, universal healthcare coverage, lifelong education systems, and well-developed digital public services have enabled socially disadvantaged groups to benefit relatively more from access to health services and digital resources. Digital inclusion policies have, to some extent, mitigated inequality risks associated with technological diffusion and digitalization processes [31–33]. In contrast, healthcare systems and digital health services in the United States and the United Kingdom rely more heavily on market-based mechanisms. Institutional fragmentation and pronounced social stratification have contributed to the concentration of digital health resources among middle- to high-income and highly educated groups [19, 34, 35]. In Global South countries such as India, South Africa, and Brazil, social inequality indicators frequently intersect with poverty, urban–rural divides, and gendered social structures. Even amid rapid smartphone adoption, vulnerable populations continue to face multiple barriers, including inadequate network quality, limited digital literacy, and affordability constraints [36, 37]. Taken together, these regional disparities suggest that the equality outcomes of digital health interventions are highly contingent upon the social policy environments in which they are embedded.

It should be noted that social inequality is not unique to digital health interventions but is a pervasive feature of health intervention delivery more broadly. Substantial disparities also exist in traditional forms of intervention, including medication access, vaccination coverage, mental health services, and in-person health education. For example, individuals with lower SES consistently lag behind in access to chronic disease medications and vaccination coverage [25, 38], while the availability of mental health services is constrained by urban–rural disparities, gaps in insurance coverage, and cultural biases [39]. Compared with traditional interventions, digital health interventions theoretically hold the potential to transcend temporal and spatial constraints and thereby enhance equality. However, in the absence of appropriate policy guidance and institutional mechanisms to safeguard digital equality, digital technologies themselves may become new barriers to health access and outcomes [21, 40]. Therefore, examining social inequality indicators in digital health interventions not only helps to uncover the latent risks associated with digital transformation but also provides critical reference points for advancing equal governance across both digital and traditional intervention domains.

Existing systematic reviews on digital health interventions and social inequality can be broadly categorized into two types: one type focuses on specific populations, such as older adults and individuals with chronic conditions, and primarily examines the barriers and risks these groups encounter in accessing, using, and accepting digital health technologies. For example, a study examining older adults’ use of digital health technologies revealed that they continue to encounter substantial barriers to accessing and utilizing such technologies. In particular, when individuals hold negative attitudes toward new technologies or lack prior usage experience, they are especially susceptible to exclusion from digital intervention systems [41]. The second category adopts a comparative perspective, examining disparities in intervention effectiveness across different socioeconomic groups. For instance, Western et al. reported that digital health interventions targeting physical activity demonstrated no significant effects among low SES groups; conversely [42], Ronteltap et al. observed positive effects of digital health interventions on dietary behaviors within similar populations [43]. Overall, although both types of reviews provide valuable insights into social inequality indicators in digital health interventions, the available evidence remains fragmented. Existing studies often focus on single populations, isolated behavioral domains, or individual dimensions of inequality, with limited systematic integration across reviews. Consequently, it remains challenging to determine whether social inequality indicators in digital health interventions exhibit cross-cutting patterns across different target behaviors, technology types, or population groups. Similarly, it is difficult to identify which vulnerable groups have been persistently underrepresented in the existing evidence base. Therefore, an umbrella review is warranted to synthesize existing research at a higher level, provide a more comprehensive evidence landscape, and elucidate underlying patterns of social inequality indicators.

Building on this rationale, this study proposes to conduct an umbrella review centered on social inequality indicators, with three specific objectives: First, to systematically organize and synthesize how existing reviews report on issues of social inequality within digital health intervention research. Second, to apply the PROGRESS-Plus framework to characterize the distribution patterns of social inequality indicators across digital health intervention reviews. Third, building on the synthesis of the above findings and informed by existing theories and relevant literature, this study examines how social inequality indicators are conceptualized and reported in current reviews, as well as their potential explanatory pathways. By achieving these objectives, this study aims to construct a comprehensive review-level evidence landscape of social inequality indicators in digital health interventions, thereby providing a theoretical foundation and empirical support for advancing equal governance in digital health.

## Methods

### Overview

This study adopts an umbrella review methodology to systematically collate and critically appraise existing systematic reviews on the use of digital health interventions for promoting adults lifestyle behavior change, with the dual aim of identifying social inequality indicators embedded within these interventions and determining which populations are being left behind. As a high-level approach to synthesizing secondary literature, umbrella reviews can consolidate the principal findings of multiple review studies, elucidate consistencies and discrepancies in the evidence base, and thereby provide a theoretical foundation for optimizing equality in digital health interventions [44].This study strictly adheres to the guidelines outlined in the PRISMA 2020 statement (Preferred Reporting Items for Systematic Reviews and Meta-Analyses) [45] and draws on research design and reporting recommendations for umbrella reviews from authoritative institutions, including the Joanna Briggs Institute (JBI) and Cochrane [44, 46], to ensure methodological transparency and the robustness of results. The study protocol has been prospectively registered with the international systematic review registry PROSPERO (registration number: CRD420251111859).

### Search strategy

This study conducted a literature search between June 1 and June 20, 2025, systematically retrieving records from seven English-language databases: Web of Science, PubMed, Scopus, Google Scholar, SPORTDiscus, ProQuest, and the Cochrane Library. The search period spanned January 2000 to June 2025. The year 2000 was selected as the starting point because it marks the period in which internet and mobile technologies began to be widely adopted in public health, and the concept of digital health interventions first emerged and gained systematic scholarly attention [47]. Studies published before 2000 primarily represented early explorations of informatization or telemedicine and differed substantially from contemporary digital health interventions in definition, delivery formats, and data infrastructures, thereby limiting their comparability. The search strategy was developed around four sets of keywords, with thematic categories combined using the Boolean operator “AND”: (1) Digital health interventions (e.g., “web-based,” “eHealth,” “mHealth,” “telehealth,” “digital health,” “mobile phone,” “social media,” etc.); (2) Lifestyle behaviors (e.g., “physical activity,” “sedentary behavior,” “sleep,” “healthy eating,” “diet,” “nutrition,” etc.); (3) Inequalities (e.g., “equity,” “inequity,” “inequality,” “disparity,” “equality,” “deprivation,” etc.); (4) Study types, including systematic reviews, meta-analyses, scoping reviews, and narrative reviews. The complete list of English-language search terms is provided in Appendix 1. To enhance the comprehensiveness of the review, manual backtracking was performed on the reference lists and cited works of all preliminarily included studies to identify additional reviews that might otherwise have been overlooked.

### Eligibility criteria

Literature screening was conducted in accordance with the PICOS framework, with specific eligibility criteria outlined as follows: (1) Study Population: Studies involving adults aged 18 years or older were included, encompassing both the general healthy population and specific groups such as individuals with chronic conditions. Minors were excluded because adolescents and children differ substantially from adults in lifestyle behaviors, digital health usage patterns, and mechanisms through which health inequality indicators emerge [48, 49]. Including these groups would have introduced substantial heterogeneity into the findings, thereby undermining the reliability of the conclusions. (2) Interventions: This study included only reviews in which digital health technologies served as the primary intervention or constituted a core component. Digital health interventions were defined as those in which key functionalities, principal behavior-change pathways, or primary data interactions relied on digital technologies for implementation. Specific formats included, but were not limited to, mobile health applications, remote consultation platforms, online behavioral intervention systems, social media–based health initiatives, virtual coaching systems, and chatbots. When digital health technologies co-occurred with other intervention modalities (e.g., face-to-face counseling, community education), reviews were included only if digital technologies constituted the primary intervention mechanism. The inclusion criteria for interventions were as follows: (i) Digital health technologies served as the primary means for delivering intervention content, feedback, monitoring, or user interaction; (ii) The mechanisms of behavior change relied on functionalities of digital technologies (e.g., automatic data capture, personalized feedback, real-time reminders); (iii) The intervention could not function effectively in the absence of digital technologies. Conversely, reviews were excluded if digital technologies were used solely for auxiliary communication, basic data recording, or when the primary intervention content was delivered offline. (3) Comparison Methodology: In the context of equality-focused research, the “Comparator” does not denote a traditional experimental control group but instead refers to comparisons across distinct social groups (e.g., place of residence, gender, income, education, race/ethnicity). Accordingly, eligible reviews could either focus exclusively on a disadvantaged population or compare disadvantaged and non-disadvantaged groups. The presence or absence of formal control groups in the primary studies included within a review did not influence eligibility for this study. (4) Outcome Measures: Eligible reviews primarily examined changes in lifestyle behaviors and associated health outcomes, with attention to the impacts or distributional differences related to social inequality indicators. In other words, lifestyle behaviors and their outcomes served as primary outcome variables, while social inequality indicators functioned as an explanatory or moderating variable. To ensure that included reviews possessed clear analytical relevance to social inequality, this study defined “explicitly addressing social inequality indicators” as follows: reviews that systematically presented, analyzed, or explained at least one social inequality indicator in their objectives, methods, results, or discussion sections. Relevant indicators included, but were not limited to, age, gender, education, income, SES, race/ethnicity, place of residence, and digital health literacy. Reviews were required to meet at least one of the following criteria: (i) Provided stratified results (e.g., reporting intervention effects by age, gender, education, income); (ii) Analyzed mechanisms of social inequality indicators (e.g., the influence of digital health literacy, device accessibility, or cultural appropriateness on intervention processes); (iii) Explicitly discussed the potential for interventions to generate or exacerbate inequality and explained the underlying mechanisms; (iv) Proposed intervention designs or policy recommendations related to social inequality. (5) Study Type: Eligible study types were limited to comprehensive review articles, including systematic reviews, meta-analyses, scoping reviews and narrative reviews, that were explicitly identified as such in their titles or abstracts.

The following types of studies were excluded: (1) Conference abstracts, dissertations, unpublished manuscripts, and other forms of gray literature were excluded because their lack of peer review and methodological transparency may compromise the comparability of research quality and the reproducibility of results; (2) Non-English publications, as restricting the search to English-language sources allows for greater methodological consistency, clearer quality control, and reduced risk of search bias. (3) Literature with overly narrow geographic or demographic scopes was excluded when it focused solely on highly specific, extremely small-scale, or uniquely contextual populations—such as single tribal communities, remote island nations, closed institutional settings (e.g., prisons), or populations restricted to specific ethnic groups, immigrant subgroups, or religious minorities. Because such populations are characterized by highly context-dependent social structures, resource conditions, and digital health usage environments, studies focusing on them cannot meaningfully represent social inequality patterns in the general adult population. Including these studies could compromise the comparability and generalizability of this study’s identification of cross-national and cross-ethnic inequality patterns; therefore, they were excluded.

### Study selection

All search results were imported into the EndNote reference management system for deduplication and assignment of identification numbers. Literature screening was conducted in two stages: title–abstract screening followed by full-text screening. Both stages were conducted independently and in parallel by two researchers (JJJ and YZH) using a double-blind review process, rather than by a single reviewer. To ensure consistency and reproducibility, the research team conducted standardized training prior to the screening process. This training included item-by-item discussion of inclusion and exclusion criteria, pilot screening exercises, and the development of standardized protocols for assessing borderline cases. During the title–abstract screening phase, both researchers independently screened all retrieved records. In the full-text screening phase, the two researchers again independently determined the eligibility of each article. Discrepancies were initially addressed through re-evaluation against the inclusion criteria; cases that remained unresolved were referred to a third researcher (ZQY), who convened a consensus meeting to determine the final decision through discussion. Furthermore, to enhance the transparency and reliability of the screening process, the research team calculated inter-rater agreement metrics during the full-text screening phase. The initial level of agreement between the two independent screeners on full-text inclusion yielded a Cohen’s kappa coefficient of 0.81, indicating a high degree of inter-rater reliability. All remaining discrepancies were ultimately resolved through consensus discussions facilitated by the third researcher.

### Data extraction and synthesis

To ensure transparency and reproducibility in data extraction, this study developed a structured extraction framework aligned with the research questions prior to formal extraction. A pilot test was conducted using six trial publications to assess the applicability of variable definitions across different types of reviews. The extraction framework included the following items: authors and publication year, review type, characteristics of the study population, type of digital health intervention, target behavior, primary outcomes, and social inequality indicators. All indicators were defined strictly on the basis of explicit reporting at the review level, without recoding or inference from primary studies. During formal extraction, two researchers (JJJ and WYF) independently reviewed each full-text article and extracted intervention methods and target behaviors strictly according to the classification systems adopted by the review authors. For instance, when a review grouped multiple technologies under categories such as “mobile health interventions” or “wearable device interventions,” this study adhered to the review’s classification without re-categorizing technologies based on descriptions in the primary studies. Similarly, the extraction of social inequality indicators was based solely on explicit reporting within the reviews, including analyses or discussions of indicators such as age, gender, education, income, SES, race/ethnicity, place of residence, employment, health condition, digital health literacy as presented in the reviews’ objectives, methods, results, or discussion sections. This study did not attempt to re-extract individual-level demographic characteristics from the primary studies cited within the reviews. Instead, all extractions were conducted solely at the review level to maintain methodological consistency and avoid cross-level inference. Furthermore, to account for differences in structure and reporting depth between narrative and scoping reviews, variables were extracted using a “tick-box” approach: a social inequality indicator was coded only when the review explicitly described, categorized, compared, or provided a rationale for it.

Disagreements between the two coders regarding the interpretation or categorization of indicators were resolved through joint review and discussion facilitated by a third researcher (ZQY) to reach consensus. Given the considerable heterogeneity among the included reviews in terms of study populations, intervention modalities, and outcome measures, this study employed a narrative synthesis approach to integrate evidence at the review level [50]. The extracted data were subsequently used to construct frequency distribution charts and dimensional distribution charts to illustrate the degree of attention given to different types of social inequality indicators within existing reviews and the corresponding evidence structures.

### Study quality assessment

This study employed review-type–specific quality assessment tools to ensure alignment between evaluation criteria and the methodological characteristics of each review design. For the included systematic reviews, AMSTAR-2 (A Measurement Tool to Assess Systematic Reviews 2) was applied to evaluate methodological quality [51]. For scoping reviews, the PRISMA-ScR (Preferred Reporting Items for Systematic Reviews and Meta-Analyses Extension for Scoping Reviews) checklist was used to assess reporting consistency and methodological transparency. For narrative reviews, the SANRA (Scale for the Assessment of Narrative Review Articles) instrument was employed to assess quality across dimensions such as transparency of the literature search, rigor of scientific argumentation, and structural coherence [52]. Quality assessments were conducted independently by two researchers (JJJ and YZH). Any discrepancies were resolved through discussion. Prior to formal evaluation, both researchers completed training and pilot scoring exercises using the AMSTAR-2, PRISMA-ScR, and SANRA guidelines to ensure consistency in the application of evaluation criteria. Given the substantial differences in research objectives, methodological requirements, and reporting standards across review types, this study did not undertake direct comparisons of quality scores among systematic reviews, scoping reviews, and narrative reviews [53]. Quality scores were not used as stratification criteria for interpreting effect differences. The primary objective of this study was to examine how social inequality indicators were conceptualized and presented within the included reviews. Accordingly, the quality assessment served primarily to gauge the reliability and transparency of the evidence base, rather than to facilitate quantitative comparisons.

### Overlap in individual studies in included systematic reviews

To evaluate the extent of overlap among the primary studies included in the systematic reviews incorporated into this umbrella review, the corrected covered area (CCA) was calculated in accordance with established methodological guidelines [54]. The CCA quantifies the proportion of overlap in the primary studies included across systematic reviews and adjusts for the number of reviews to minimize bias arising from variations in review count. The CCA value of 100% indicates that all systematic reviews include exactly the same primary studies (complete overlap), whereas a value of 0% indicates that the primary studies included in each systematic review are entirely distinct (no overlap).

The CCA values were interpreted according to the following thresholds: (1) < 5% = extremely low overlap; (2) 6%-10% = moderate overlap; (3) 11%-15% = high overlap; and (4) > 15% = very high overlap. Higher CCA values indicate substantial evidence overlap among systematic reviews, potentially reducing the independence and novelty of this umbrella review’s synthesized results. Conversely, lower CCA values suggest greater distinctiveness among the primary studies, thereby enhancing the diversity and complementarity of the synthesized findings.

This study included systematic reviews, meta-analyses, scoping reviews, and narrative reviews during the initial literature screening phase. However, scoping reviews and narrative reviews were not subjected to CCA calculations in the subsequent quantitative analyses. Their inclusion in the screening process was guided by the following methodological considerations: First, although scoping and narrative reviews differ from systematic reviews in research objectives and methodological logic, they perform a critical “evidence-mapping” function within digital health inequality research. They systematically present the current state of research, key thematic areas, and evidence gaps within a given field. For example, scoping reviews typically map the breadth of research topics rather than synthesizing primary studies in a rigorous manner, whereas narrative reviews contribute essential elements such as theoretical frameworks, conceptual mechanisms, and developmental trajectories of the field. Including these review types therefore facilitates a more comprehensive understanding of inequality structures within digital health interventions. Second, a core objective of this study is to analyze how existing reviews present and discuss social inequality. Under this objective, the reviews themselves constitute the primary units of analysis, rather than serving merely as sources from which to extract results from the underlying primary studies. In other words, this study focuses on the presentation of inequality at the review level rather than on the intervention effects reported in the original studies contained within them. Thus, irrespective of whether a review contains primary studies suitable for quantitative synthesis, its conceptual discussions, problem identification, and explanatory mechanisms relating to social inequality remain of substantive analytical value. Third, CCA (Corrected Covered Area) is designed to assess the degree of overlap among primary studies included in systematic reviews and is therefore a method specifically intended for quantitative evidence derived from systematic reviews. Scoping and narrative reviews often incorporate large bodies of literature spanning multiple disciplines and methodologies, including diverse forms of evidence such as qualitative studies, policy reports, and expert opinions. Including such reviews in CCA calculations would distort results owing to substantial heterogeneity and would fail to reflect genuine overlap among primary studies. Consequently, CCA calculations were applied only to eligible systematic reviews, whereas scoping and narrative reviews were excluded from this stage of analysis. In summary, although the inclusion of scoping and narrative reviews enhances the theoretical understanding and evidence landscape surrounding social inequality in digital health, these review types were excluded from subsequent quantitative analyses of primary-study overlap in order to maintain methodological rigor.

### Coding and classification

To ensure transparency and reproducibility in the extraction and classification of social inequality indicators, this study adopts the PROGRESS-Plus framework as the primary classification framework during both data extraction and results synthesis [55]. The PROGRESS-Plus framework is a widely used analytical tool in international health equality research, designed to systematically identify key social structural indicators shaping disparities in health opportunities and intervention benefits. It encompasses indicators including place of residence, race/ethnicity, employment, gender, education, SES. Prior to coding implementation, the research team predefined the scope and typical expressions associated with each social inequality indicator in accordance with the PROGRESS-Plus framework. It was agreed that when a single review addressed multiple social inequality indicators, a multiple-coding strategy would be applied, whereby each indicator was identified and recorded separately without forcing consolidation into a single category. The coding process comprised three sequential steps. The first step involved the identification of social inequality indicators. The research team conducted full-text screening of all included reviews, with particular attention to key sections such as the results and discussion, to systematically identify content related to social inequality indicator. Identified indicators included both explicit demographic indicators used for comparative analyses (e.g., age, gender, income, education, place of residence, race/ethnicity) and implicit disparities reflected in descriptions of intervention accessibility, participation barriers, usage burden, or differential responses. For such implicit expressions, coding decisions were determined based on the primary social structural attribute indicated. For example, descriptions of device affordability or economic accessibility were coded under the income indicator, whereas accounts of comprehension difficulties or digital operational barriers were assigned to the digital health literacy indicator. The second step involved classification mapping. The research team mapped each social inequality identified in Step 1 to its corresponding indicator within the PROGRESS-Plus framework and archived the coding results. When a statement potentially involved multiple social inequality indicators, the primary code was determined based on the principal explanatory pathway emphasized by the review authors. If a statement served multiple explanatory functions, a secondary code was assigned in addition to the primary code to reduce potential omissions. The third step involved unit coding. After identifying and classifying social inequality indicators, analytical units were constructed based on the combination of “social inequality indicator × target lifestyle behavior domain.” Target lifestyle behavior domains were uniformly categorized into four types based on the scope of included reviews: physical activity, sedentary behavior, sleep, and diet. When a single review addressed multiple behavior domains, each domain was assigned to its corresponding analytical unit. For each analytical unit, the research team applied rule-based coding to characterize manifestations of social inequality indicators in digital health interventions using a standardized symbol system: The symbol (–) indicates that socially disadvantaged groups exhibit lower levels of accessibility, participation, adherence, or outcome improvement in digital health interventions; (+) indicates that socially disadvantaged groups achieve intervention benefits comparable to or exceeding those of more advantaged groups; (0) indicates no substantively meaningful differences observed between social groups; (*) indicates inconsistent or mixed evidence across studies or intervention types. Directional judgments were based on overarching conclusions drawn from systematic reviews rather than individual study findings, thereby reducing the risk of misinterpretation arising from study-level variation. Concurrently, to characterize how social inequality indicators manifest in digital health interventions, the research team synthesized narrative explanations across analytical units to identify recurring “typical manifestations.” Specifically, the study extracted barriers, mechanisms, and contextual explanations relevant to each analytical unit from the results and discussion sections of included reviews (e.g., insufficient digital literacy, limited device or network accessibility, time and caregiving pressures, unstable work schedules, and technology designs that inadequately consider specific group needs). Semantically similar expressions within the same analytical unit were merged and generalized. Only when an explanatory statement recurred across multiple independent reviews was it generalized as a typical manifestation for the corresponding analytical unit. This approach ensured that identified typical manifestations reflected cross-review commonalities rather than isolated phenomena observed in individual studies.

## Results

### Study characteristics

**Fig. 1:**
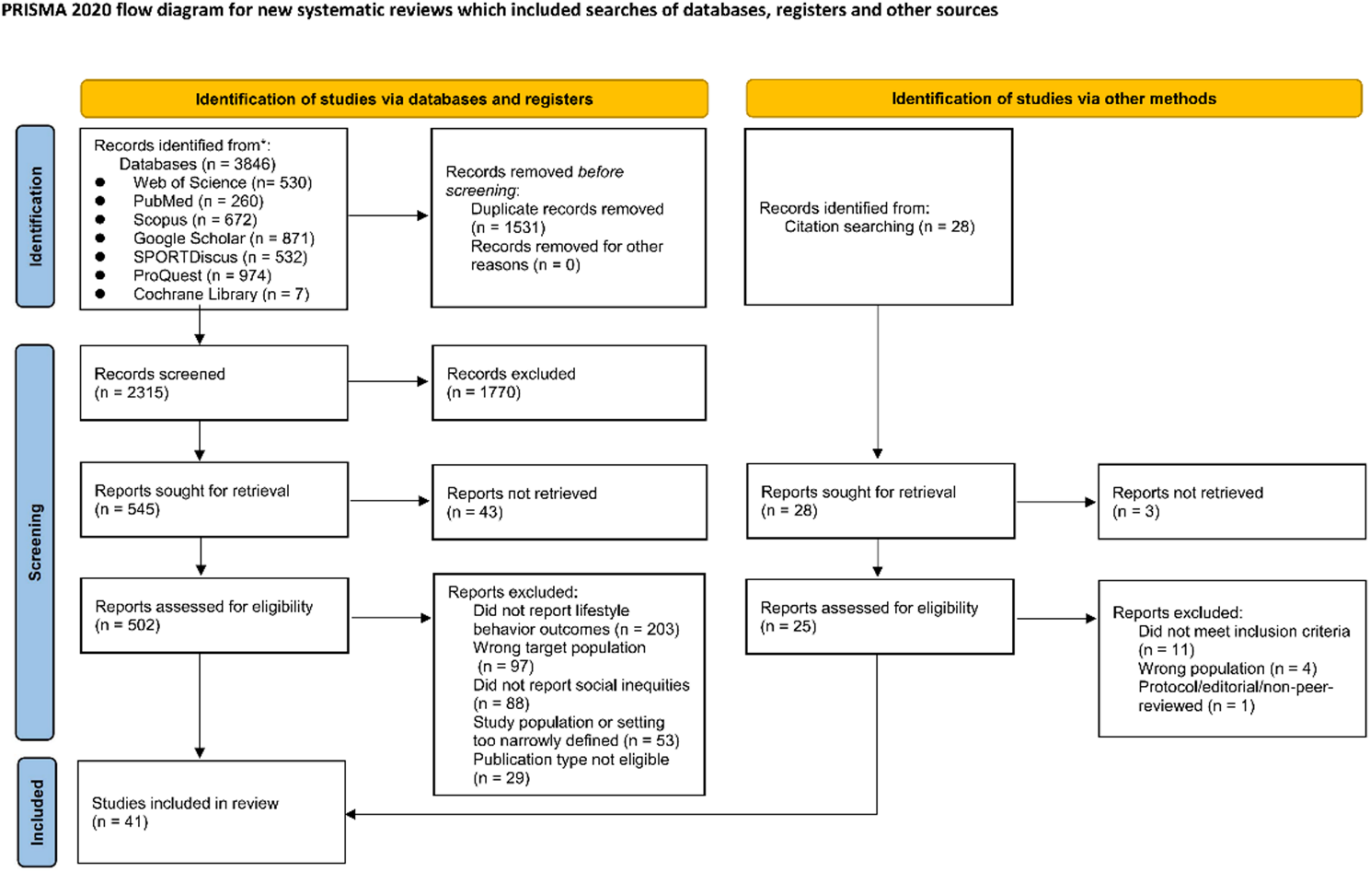
PRISMA Flow Diagram of the Study Selection Process

This study retrieved a total of 3,846 literature records from seven English-language databases. After automated comparison using literature management software and manual verification, 1,531 duplicate records were removed, leaving 2,315 records to proceed to the initial screening phase based on titles and abstracts. After deduplication and initial screening, 502 records proceeded to full-text review. Based on the predefined exclusion criteria, the following records were removed: those not reporting lifestyle behavior outcomes (*n* = 203, 40%); wrong target population (*n* = 97, 19%); those not reporting social inequality indicators (*n* = 88, 18%); those with overly narrow geographical or ethnic restrictions (*n* = 53, 11%); and those not meeting the inclusion criteria for study type (*n* = 29, 6%). Manual backward citation searches of the references and cited literature of the included studies identified nine additional review articles. In total, 41 reviews met the inclusion criteria and were incorporated into the final analysis (Fig. 1).

The publication years of the included reviews ranged from January 2000 to June 2025, with 34 (83%) published after 2019, indicating rapid growth in this field in recent years (Table 1). The number of primary studies included in each review ranged from 4 to 231, reflecting substantial heterogeneity in study size. The study populations encompassed college students, general adult populations, older adults, pregnant women and new mothers, individuals with chronic diseases, and other socially vulnerable groups. It is important to note that individuals younger than 18 years of age were excluded based on this study’s inclusion criteria; consequently, all primary studies synthesized within the reviews involved adult participants. The populations presented here refer to the sample characteristics of the primary studies synthesized by each review, rather than the population scope defined by the reviews themselves. The purpose is to illustrate the distribution and coverage disparities of existing digital health intervention evidence across different adult populations. Intervention targets primarily addressed improving physical activity levels (*n* = 37, 90%) [13, 14, 16, 17, 19, 42, 56–86], followed by improving diet (*n* = 12, 29%) [56–58, 64, 73, 80, 81, 87–91], reducing sedentary behavior (*n* = 7, 17%) [19, 57, 60, 61, 66, 70, 85], and improving sleep (*n* = 6, 15%) [19, 57, 67, 71, 92, 93]. 19 reviews examined integrated interventions targeting multiple lifestyle behaviors [14, 16, 19, 56–58, 60, 61, 64, 66, 70, 71, 73, 76, 80, 81, 85, 91, 92]. With respect to intervention modalities, this study categorizes digital health interventions into five types based on delivery platforms and functional characteristics, drawing on the WHO’s Universal Classification of Digital Health Interventions [94]: (1) Web-based platforms and online systems (*n* = 25, 61%): including online learning modules, interactive web-based tasks, and health behavior tracking systems; (2) Mobile applications (*n* = 22, 54%): typically integrating goal setting, feedback mechanisms, reminder functions, and behavior-monitoring capabilities; (3) Communication- and messaging-based interventions (*n* = 28, 68%): including telephone-based interventions (*n* = 18) and SMS/text messaging interventions (*n* = 10), primarily used for behavioral reminders, follow-up support, and personalized communication; (4) Wearable monitoring devices (*n* = 8, 20%): such as pedometers, accelerometers, and smart bracelets, used for continuous monitoring of physical activity and related physiological indicators; (5) Social and intelligent interaction tools (*n* = 12, 29%): including social media platforms (*n* = 5), handheld digital devices (*n* = 3), video-based interventions (*n* = 3), chatbots (*n* = 1), and virtual assistants (*n* = 1), primarily used to enhance interactivity, motivate user engagement, or provide personalized communication support, particularly for socially vulnerable groups. Overall, digital health interventions predominantly rely on web-based platforms, mobile applications, and communication-oriented approaches, each representing more than half of the total interventions identified. In contrast, wearable devices and social or intelligent interaction technologies appear less frequently, often functioning as supplementary components or emerging modalities.

**Table 1:** Characteristics of included reviews

Author, Year	Type of Review	Focus	Studies	*N*	Intervention(s)	Target behavior(s)	Inequality indicator(s)
Arambepola et al., 2016 [56]	systematic review	patients with type 2 diabetes	15	1155	automated brief messages	diet, physical activity	place of residence
Badr et al., 2024 [57]	scoping review	adults	41	NR	computer, web, text message, mobile phone, email	diet, sleep, physical activity, sedentary behavior	age, gender/sex, race/ethnicity, education, SES, income, medical condition
Baer et al., 2022 [58]	systematic review	middle-aged and older adults	7	8870	smartphone apps	diet, physical activity	age
Bennett et al.,2014 [59]	systematic review	racial/ethnic minority adults	6	4899	computer, web, text, mobile phone, apps, email	physical activity	race/ethnicity
Bi et al.,2024 [16]	systematic review and meta-analysis	college students	8	569	mobile phones, tablets, web-based interventions, SMS, social media, lifestyle or fitness smartphone apps, and wearable devices.	light physical activity, moderate to vigorous physical activity, sedentary time, steps.	place of residence
Boima et al., 2024 [13]	systematic review and meta-analysis	patients with hypertension	22	12,892	smartphone app, SMS mobile phone call	physical activity	place of residence
Buckingham et al., 2019 [60]	systematic review	adults	25	73,440	mobile phone, smartphone apps, PDAs, tablets, wearable activity, monitors/trackers	physical activity, sedentary behavior	age, gender/sex, place of residence,
Cheng et al., 2020 [61]	systematic review	socially disadvantaged groups	48	8105	web-based intervention	physical activity, sedentary behavior	SES, digital health literacy
Clark et al., 2023 [62]	systematic review	adults	9	1606	computer, web, text message, mobile phone, email	physical activity	income
Davis et al., 2020c [63]	systematic review	adults	16	1972	tailored mHealth interventions	physical activity	income
Duan et al., 2021 [64]	systematic review and meta-analysis	people with noncommunicable diseases	15	NR	web sites or pages, telephone counseling, text messaging	diet, physical activity	age, gender/sex
Duff et al., 2017 [65]	systematic review	people with cardiovascular disease	23	3500	Internet, web-based program, mobile phone, telemonitoring	physical activity	age, gender/sex
Elavsky et al., 2019 [66]	systematic review	older adults	52	5882	cell phones, text messaging, and mobile apps	sleep, physical activity, sedentary behavior	age
Enyioha et al., 2022 [67]	systematic review	adults	7	942	cellular phone calls, text messaging, web-based apps, mobile apps	physical activity	race/ethnicity
Greco et al., 2023 [17]	narrative review	older adults	21	1786	web-based interventions, virtual game-based intervention	physical activity	age, gender/sex
Gravesande et al.,2023 [68]	scoping review	older adults	18	715	videoconferencing systems, DVDs, videolinks, videos websites	physical activity	age
Joseph et al., 2019 [69]	systematic review	african american and hispanic women	10	860	mobile phone or smartphone, social media platform, internet-based website, e-mail, or text messaging	physical activity	race/ethnicity, gender/sex
Khoo et al., 2021 [70]	systematic review	cancer survivors	31	1977	web-based apps, email, text messages, website, activity trackers	physical activity, sedentary behavior	place of residence
Lau et al., 2020 [87]	systematic review and meta-analysis	overweight adults	15	5816	mobile apps, websites, web-based program, text messaging, technology-based systems, social media, wearable devices, video calls	diet	age, gender/sex
Lee et al., 2023 [71]	scoping review	patients with cancer	231	NR	web-based intervention	physical activity, sleep	age, disease
Liu et al., 2020 [72]	systematic review and meta-analysis	older adults	10	1035	wearable activity tracker	physical activity	age
Leonard et al., 2021 [73]	systematic review and meta-analysis	pregnant women	21	6265	tracking tools incorporating devices daily	diet, physical activity	gender/sex
Livingstone et al.,2023 [88]	systematic review	adults	30	13,365	smartphone apps, text messages, websites, phone calls, emails	diet	age, place of residence
Lu et al., 2025 [93]	systematic review and meta-analysis	college students	13	5251	virtual therapist	sleep	gender/sex
Mclaughlin et al.,2021 [14]	systematic review and meta-analysis	adults	19	7876	mobile apps, web-based intervention, smartphone	physical activity, sedentary behaviour	age, gender/sex, income
Myers-Ingram et al.,2023 [75]	systematic review	adults	4	373	web-based, mobile apps, text, social media	physical activity	income, education, occupation/employment
Peng et al., 2023 [76]	systematic review and meta-analysis	college students	9	527	smartphone apps, wearable activity trackers (pedometers or accelerometers), social media, online websites	physical activity, sedentary behaviour	gender/sex
Qiu et al., 2018 [77]	systematic review and meta-analysis	patients with chronic obstructive pulmonary disease	15	1316	step-counters, mobile apps,	physical activity	age, gender/sex
Robert et al.,2021 [89]	systematic review and meta-analysis	middle-aged and older adults	70	19,400	mobile apps, web-based app, phone calls	diet	age
Schoeppe et al.,2016 [91]	systematic review	adults	27	2969	apps, web	diet, physical activity, sedentary behaviour	age, gender/sex
Sequi-Dominguez et al.,2020 [79]	systematic review and meta-analysis	adults with metabolic syndrome	9	709	mobile apps, phone calls, web	physical activity	age
Singh et al., 2024 [92]	systematic review	adults	47	20,687	chatbots, web	sleep, physical activity, fruit and vegetable consumption	SES, digital health literacy
Sherifal et al.,2017 [80]	systematic review and meta-analysis	pregnant and postpartum women	10	525	common eHealth technological elements	diet, physical activity	gender/sex
Szinay et al., 2023 [81]	systematic review	adults	16	290,039	smartphone, PDAs, wearable activity tracker	diet, physical activity	age, education, race/ethnicity, gender/sex, income, location, occupation, employment
Yao et al., 2022 [19]	scoping review	adults	41	NR	common eHealth technological elements	sleep, physical activity, sedentary behavior	age, race/ethnicity, region, SES, and education level, together with health conditions and eHealth literacy
Yang et al., 2022 [82]	scoping review	adults	54	8975	smartphones, smartwatches, PDAs, wristbands, and other wireless technologies	physical activity	gender/sex, age, education
Western et al.,2021 [42]	systematic review and meta-analysis	adults	19	5419	smartphone apps, text messages, websites	physical activity	SES
Wong et al., 2022 [83]	systematic review and meta-analysis	overweight adults	30	5391	wearable devices, accelerometers, pedometers, tailored wearable systems	physical activity	location, age
Wongvibulsin et al., 2021 [84]	systematic review	people with cardiac rehabilitation	31	7608	telephone calls, text messages, apps	physical activity	digital health literacy
Wu et al., 2023 [86]	systematic review and meta-analysis	older adults	45	7144	wearable activity trackers	physical activity, sedentary behavior	age
Zheng et al., 2023 [90]	systematic review and meta-analysis	adults	34	5691	mobile apps, short-message services, and mobile device-compatible websites	diet	place of residence, SES

Furthermore, among the 41 included reviews, the majority acknowledged social inequality but did not systematically define or conceptualize it as a central analytical construct. Only five reviews (12%) explicitly articulated conceptual frameworks or theoretical foundations for social inequality [19, 42, 57, 61, 81], for example by drawing on established frameworks such as PROGRESS-Plus. In contrast, 36 reviews (88%) approached inequality primarily at a descriptive level, for example by comparing intervention outcomes across population groups, without engaging in systematic conceptualization or theory-driven categorization.

### Quality assessment results

The methodological quality assessment results for each review type are summarized in Appendix 2. This study included 41 reviewss, of which systematic reviews were appraised using AMSTAR-2 (*n* = 35, 85%), scoping reviews were evaluated according to the PRISMA-ScR guidelines (*n* = 5, 12%), and narrative reviews were assessed using the SANRA tool (*n* = 1, 3%). Among the 35 systematic reviews assessed with AMSTAR-2, 19 were rated as very low confidence, 9 as low confidence, 5 as moderate confidence, and only two were classified as high confidence. The most common contributors to low confidence ratings included failure to address risk of bias within the discussion (*n* = 19), absence of assessment or interpretation of publication bias and its potential implications (*n* = 13), and insufficient examination of heterogeneity among included studies (*n* = 7). Additionally, the narrative review included in this study (*n* = 1) received a relatively higher SANRA score, although limitations persisted in retrieval transparency and the clarity of scientific justification. With respect to the PRISMA-ScR assessment of the five scoping reviews, four demonstrated satisfactory performance in reporting transparency, methodological process description, and justification of data selection, while variability remained in the reproducibility of search strategies and the completeness of evidence presentation.

### Overlap in individual studies in systematic reviews

Across the 41 reviews included in this umbrella review, a total of 771 primary studies meeting the inclusion criteria were identified. After deduplication, 646 unique primary studies cited across the 41 reviews were retained. The calculated CCA was 0.57%, indicating a low degree of overlap among the primary studies, classified as minimal overlap. The detailed calculation process for the CCA is provided in Appendix 3.

### Social inequality indicators reporting

**Fig. 2:**
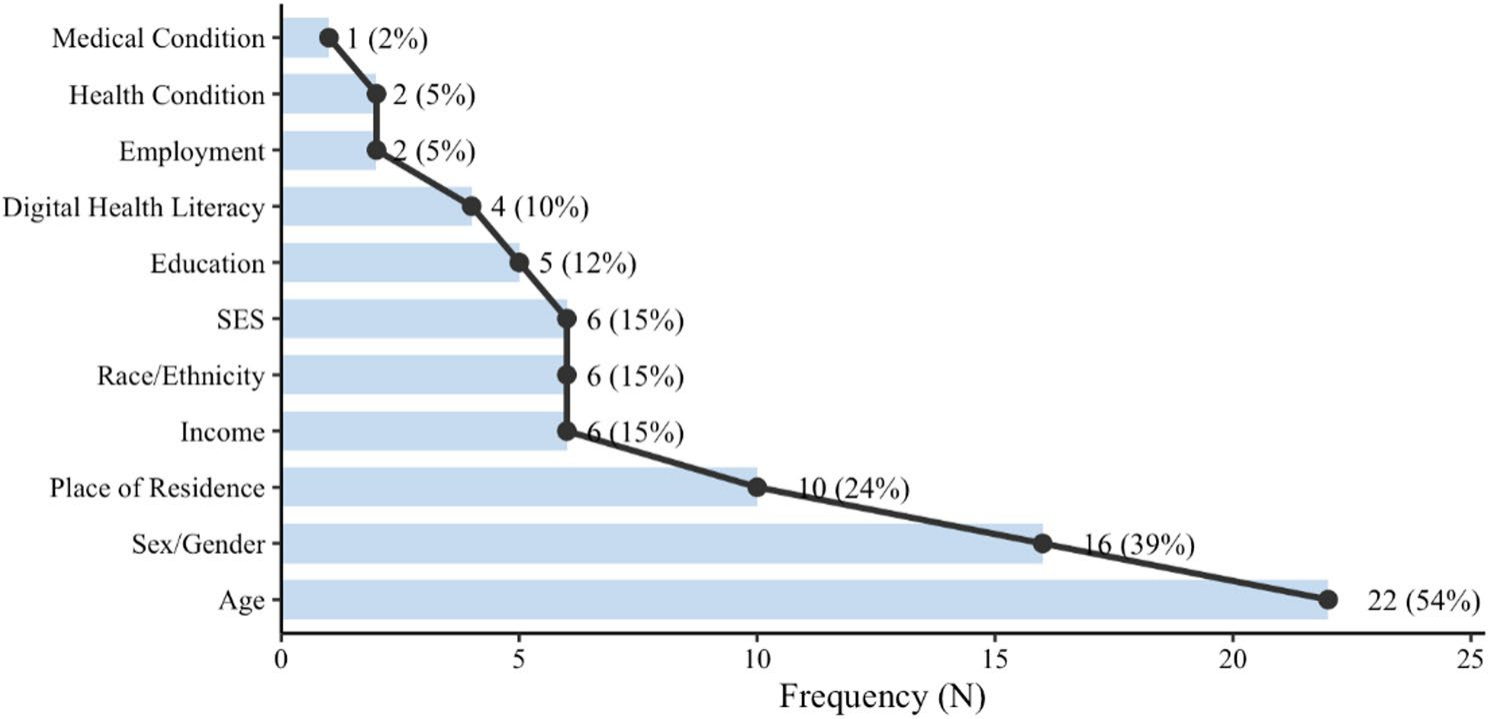
Social Inequality Indicators Interpreted through the PROGRESS-Plus Framework

Based on a systematic inductive coding process guided by the PROGRESS-Plus framework, this study identified 11 distinct categories of social inequality indicators (see Fig. 2). Specifically, the identified indicators included age (*n* = 22, 54%) [14, 17, 19, 57, 58, 60, 64–66, 68, 71, 72, 77, 79, 81–83, 85, 87–89, 91], gender (*n* = 16, 39%) [14, 17, 57, 60, 64, 65, 69, 73, 76, 77, 80–82, 87, 91, 93], place of residence (*n* = 10, 24%) [13, 16, 19, 56, 60, 70, 81, 83, 88, 90], income (*n* = 6, 15%) [14, 57, 62, 63, 75, 81], race/ethnicity (*n* = 6, 15%) [19, 57, 59, 67, 69, 81], SES (*n* = 6, 15%) [19, 42, 57, 61, 90, 92], education (*n* = 5, 12%) [19, 57, 75, 81, 82], digital health literacy (*n* = 4, 10%) [19, 61, 84, 92], employment (*n* = 2, 5%) [75, 81], health condition (*n* = 2, 5%) [19, 71], and medical condition (*n* = 1, 2%) [57]. Overall, age, gender, and place of residence emerged as the three most frequently reported social inequality indicators across the included reviews. By contrast, income, race/ethnicity, and education received comparatively less analytical attention. Furthermore, digital health literacy, employment, health condition, and medical condition were only sporadically addressed, often limited to supplementary descriptions or heterogeneity analyses, and rarely subjected to systematic or theory-informed examination (see Table 2).

**Table 2:** Social inequality indicators reported in included reviews

Indicators	*N*	Target Behavior	Direction	Typical Manifestations	Results	Supporting Reviews	Over All
Age	22	PA	(+)	Some studies found that older adults achieved comparable or even higher digital intervention benefits than younger adults, particularly when interventions incorporated automated guidance or simplified wearable devices.	4/19 (16%)	[19, 82, 83, 85]	(-)
			(-)	Most studies reported that older adults had lower participation, poorer adherence, or reduced responsiveness in PA-related digital interventions due to limited digital skills, lower device familiarity, and accessibility barriers.	12/19 (68%)	[14, 17, 57, 58, 60, 65, 66, 68, 71, 72, 77, 79]	
			(0)	One review reported no meaningful differences between older and younger adults in PA-related digital intervention outcomes.	1/19 (5%)	[64]	
			(*)	Some reviews reported inconsistent findings across included studies, with older adults showing both high and low levels of engagement depending on intervention type and usability features.	2/19 (11%)	[81, 91]	
		SB	(-)	Older adults frequently showed lower participation or reduced effectiveness in SB-related digital interventions due to lower digital literacy and device usability barriers.	5/7 (71%)	[14, 19, 57, 60, 66]	(-)
			(+)	One review reported that older adults performed better or showed greater SB improvements when interventions incorporated simple wearable devices or automated feedback.	1/7 (14%)	[85]	
			(*)	One review reported mixed findings regarding SB-related digital engagement among older adults, with outcomes varying by intervention design.	1/7 (14%)	[91]	
		Sleep	(-)	Older adults tended to have poorer digital access or lower engagement with sleep-related digital tools, limiting their ability to benefit from these interventions.	3/4 (75%)	[19, 57, 66]	(-)
			(0)	One review reported no meaningful age-related differences in the use or outcomes of sleep-related digital interventions.	1/4 (25%)	[71]	
		Diet	(-)	Older adults generally showed lower digital access and reduced engagement in diet-related digital programs compared with younger adults.	5/8 (63%)	[57, 58, 88, 89, 91]	(-)
			(0)	One review reported no significant age-related differences in diet-related digital health outcomes.	2/8 (25%)	[64, 87]	
			(*)	Within a single review, diet-related digital engagement among older adults showed mixed patterns, with outcomes varying across interventions.	1/8 (12%)	[81]	
Gender/ Sex	16	PA	(-)	Women often showed lower engagement or poorer outcomes in PA-related digital interventions, largely due to competing caregiving responsibilities, lower digital confidence, or limited time resources.	7/14 (50%)	[14, 17, 60, 64, 73, 76, 82]	(-)
			(+)	One review suggested that women, particularly African American and Hispanic women, may benefit more from PA-related digital programs when interventions focus on weight management and social support.	1/14 (7%)	[69]	
			(*)	Some studies reported inconsistent gender differences in PA outcomes, with effects varying across intervention features and digital modalities.	2/14 (14%)	[57, 81]	
			(0)	Some studies reported no meaningful gender differences in PA-related digital intervention outcomes.	4/14 (29%)	[65, 77, 80, 91]	
		SB	(-)	Across all available evidence, women demonstrated lower access or less consistent engagement in SB-related digital health interventions compared with men.	5/5 (100%)	[14, 57, 60, 76, 91]	(-)
		Sleep	(-)	One review indicated that women showed lower digital access or poorer engagement in sleep-related interventions.	1/2 (50%)	[93]	(-)
			(*)	One review showed inconsistent gender differences in sleep-related digital intervention outcomes, with effects varying across tools and populations.	1/2 (50%)	[57]	
		Diet	(-)	Women frequently demonstrated lower dietary intervention engagement, often due to barriers such as digital access limitations or competing household responsibilities.	4/7 (57%)	[57, 73, 80, 91]	(-)
			(0)	Several studies found no significant gender differences in digital diet-related outcomes.	3/7 (43%)	[64, 81, 87]	
Place of Residence	10	PA	(-)	Residents in rural or remote areas generally showed lower digital access and poorer participation in PA-related digital interventions, primarily due to limited broadband availability, lower device ownership, and reduced exposure to digital health technologies.	6/8 (75%)	[13, 16, 19, 60, 70, 83]	(-)
			(+)	One review suggested that rural participants benefited from PA-related digital interventions when low-bandwidth or SMS-based components were used, which reduced technological barriers.	1/8 (12.5%)	[56]	
			(*)	One review reported mixed findings regarding rural–urban differences, with patterns varying across intervention types and digital delivery formats.	1/8 (12.5%)	[81]	
		SB	(-)	Across all available studies, rural residents consistently displayed poorer digital access and lower engagement with sedentary behavior-related digital health interventions compared with urban residents.	4/4 (100%)	[16, 19, 60, 70]	(-)
		Sleep	(-)	Rural residents showed lower digital access and reduced participation in sleep-related digital health tools compared with their urban counterparts.	1/1 (100%)	[19]	(-)
		Diet	(-)	Diet-related evidence consistently showed that rural residents had lower digital access and reduced use of online diet programs compared with urban populations, reflecting structural disparities in connectivity and device availability.	4/4 (100%)	[56, 81, 88, 90]	(-)
Income	6	PA	(-)	Low-income adults consistently demonstrated lower digital access and poorer engagement in PA-related interventions due to barriers such as limited smartphone ownership, data plan affordability, and reduced familiarity with digital health tools.	5/6 (83%)	[14, 57, 62, 63, 75]	(-)
			(*)	One review reported inconsistent income-related differences in PA outcomes, with effects varying depending on intervention format and accessibility features.	1/6 (17%)	[81]	
		SB	(-)	Sedentary-behavior interventions consistently showed lower engagement among low-income groups, reflecting persistent digital access and affordability barriers.	2/2 (100%)	[14, 57]	(-)
		Diet	(*)	Evidence for diet-related interventions was mixed, with some studies reporting inconsistent income-related differences.	1/1 (100%)	[81]	(*)
Race/ Ethnicity	6	PA	(-)	Racial and ethnic minority groups-such as African American, Hispanic, and American Indian populations-consistently showed lower digital access and weaker participation in PA-related digital interventions than non-Hispanic White adults.	3/6 (50%)	[19, 57, 59]	(-)
			(*)	Evidence for PA-related outcomes among minority racial/ethnic groups was mixed, with some studies reporting significant improvements while others found inconsistent or limited effects.	3/6 (50%)	[67, 69, 81]	
		Sleep	(-)	Sleep-related evidence uniformly showed that minority racial/ethnic groups had reduced digital access and lower engagement compared with White adults.	2/2 (100%)	[19, 57]	(-)
SES	6	PA	(-)	Individuals with lower SES consistently showed reduced digital access, lower engagement, or poorer outcomes in PA-related interventions due to financial constraints, limited device ownership, and lower digital literacy.	4/5 (80%)	[19, 57, 61, 92]	(-)
			(*)	One review indicated inconsistent SES differences in PA outcomes, with intervention effects varying across delivery formats and user contexts.	1/5 (20%)	[42]	
		SB	(-)	Lower-SES groups exhibited uniformly poorer digital access and lower engagement in SB-related interventions.	3/3 (100%)	[19, 57, 61]	(-)
		Sleep	(-)	Across all studies, individuals with lower SES showed poorer digital access and reduced participation in sleep-related digital health programs.	3/3 (100%)	[19, 57, 92]	(-)
		Diet	(-)	Diet-related interventions consistently revealed lower engagement and poorer digital access among low-SES participants.	3/3 (100%)	[57, 90, 92]	(-)
Education	5	PA	(-)	Adults with lower educational attainment showed reduced digital health literacy, difficulty interpreting digital feedback, and lower engagement in PA-related digital interventions.	4/5 (80%)	[19, 57, 75, 82]	(-)
			(0)	One review reported no significant differences in PA-related digital outcomes between higher- and lower-education groups.	1/5 (20%)	[81]	
		Sleep	(-)	Sleep-related evidence uniformly indicated lower digital literacy and poorer engagement among adults with lower educational attainment.	2/2 (100%)	[19, 57]	(-)
		Diet	(0)	One review found no clear education-related differences in diet-related digital health outcomes.	1/1 (100%)	[81]	(0)
Digital Health Literacy	4	PA	(-)	Individuals with lower digital health literacy consistently showed reduced ability to navigate digital platforms, interpret feedback, or sustain participation in PA-related digital interventions.	4/4 (100%)	[19, 61, 84, 92]	(-)
		SB	(-)	Lower digital health literacy was associated with poorer engagement in SB-related digital interventions due to limited skills in device use and information processing.	2/2 (100%)	[19, 61]	(-)
		Sleep	(-)	Adults with low digital health literacy had reduced participation and lower responsiveness to sleep-related digital health tools.	2/2 (100%)	[19, 92]	(-)
Occupation/ Employment	2	PA	(-)	Individuals with irregular, shift-based, or unstable employment patterns showed lower engagement in PA-related digital interventions due to variable schedules and limited time.	2/2 (100%)	[75, 81]	(-)
Health Condition	2	PA	(-)	Individuals with poorer physical or mental health demonstrated reduced capacity to use PA-related digital tools effectively, particularly when accessibility features were insufficient.	2/2 (100%)	[19, 71]	(-)
		Sleep	(-)	Lower health status was associated with poorer use of sleep-related digital interventions, reflecting both functional limitations and reduced digital readiness.	2/2 (100%)	[19, 71]	(-)
Medical Condition	1	PA	(-)	Individuals with chronic or complex medical conditions often required more tailored or intensive digital support, leading to reduced engagement in standard PA-related digital interventions.	1/1 (100%)	[57]	(-)

### Group differences in digital health intervention effects

Digital health interventions exhibit heterogeneous effects across population subgroups, reflecting differential responsiveness to intervention design and delivery. With respect to age, several reviews suggest that older adults can derive measurable benefits from digital health interventions, particularly in terms of increasing physical activity levels [19, 82, 83, 85] and reducing sedentary behavior [85]. However, other reviews consistently report that older adults encounter substantial barriers related to technology use, interface adaptability, and sustained adherence, resulting in significantly lower intervention participation rates compared with middle-aged and younger adults [14, 17, 57, 58, 60, 65, 66, 68, 71, 72, 77, 79]. With respect to gender, most reviews report that male participants demonstrate more favorable responses to physical activity-focused digital interventions [14, 17, 60, 64, 73, 76, 82]. In contrast, some reviews observe that female participants report lower confidence in engaging with digital health platforms, which is associated with reduced intervention acceptance and persistence [95]. However, this pattern is not consistently observed across studies; for example, Szinay et al. reported stronger adherence among female in two included studies [81]. With respect to SES, individuals with lower levels of education, income, and overall SES experience heightened technological barriers, including limited access to devices, insufficient digital health literacy, and gaps in relevant knowledge [19, 82, 84, 92]. Consequently, intervention uptake, adherence, and overall effectiveness among these populations are consistently lower than those observed in middle- and high-income groups [14, 62, 63, 75]. With respect to place of residence, several reviews highlight that in developing countries and remote regions, the implementation of digital health interventions is substantially constrained by weak network infrastructure [90], limited access to terminal devices [57, 83], language mismatches, and insufficient cultural adaptation of intervention content and formats [13, 56]. As a result, rural residents demonstrate markedly lower levels of intervention participation and derived benefits compared with their urban residents [60, 88].

### Target behaviors and social inequality indicators

**Fig. 3:**
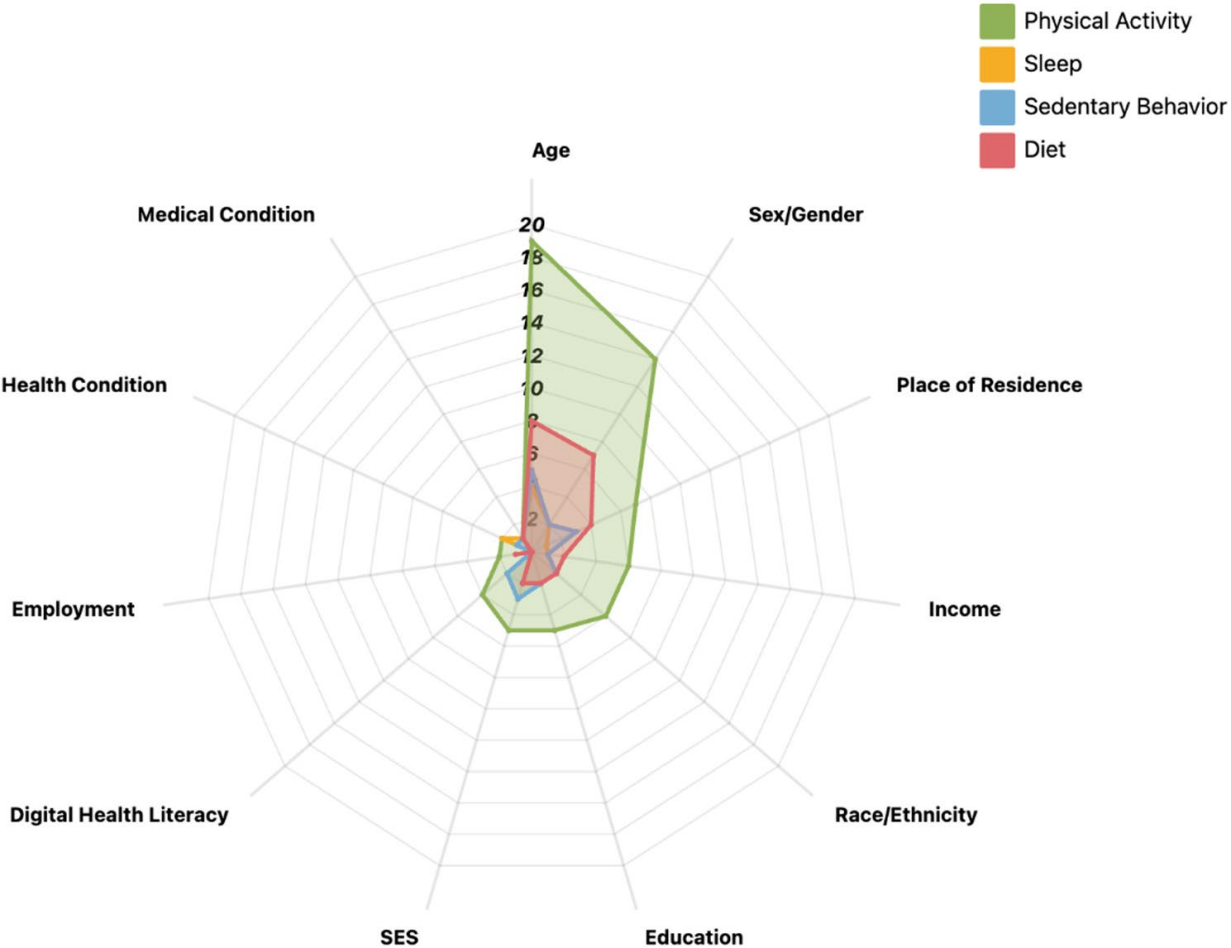
Target Behaviors and Social Inequality Indicators

Substantial variation is observed in how social inequality indicators are addressed across different target behavioral domains (see Fig. 3). Physical activity interventions most frequently incorporate explicit analyses of social inequality indicators, with a particular focus on disparities related to age, gender, education, and place of residence. In addition, some reviews also address the impact of income and broader SES. Within these studies, older adults, individuals with lower education, and low-income populations consistently exhibit poorer outcomes in terms of intervention accessibility, adherence, and long-term effectiveness [19, 82, 84, 92]. Reviews of dietary interventions primarily highlight disparities associated with income, education, and race/ethnicity, with individuals from higher-income and higher-education groups more likely to adopt healthy eating recommendations [57, 58, 88, 89, 91, 96]and to sustain higher levels of engagement with digital dietary management platforms. In contrast, reviews examining sedentary behavior and sleep interventions remain limited in number, and most do not position social inequality indicators as a core analytical dimension. Only sporadic references note disadvantages in participation and intervention benefits among groups such as older adults and rural residents [19, 71, 90], thereby revealing substantial gaps in equality-focused research within these behavioral domains.

### Target behaviors and digital health technologies

**Fig. 4:**
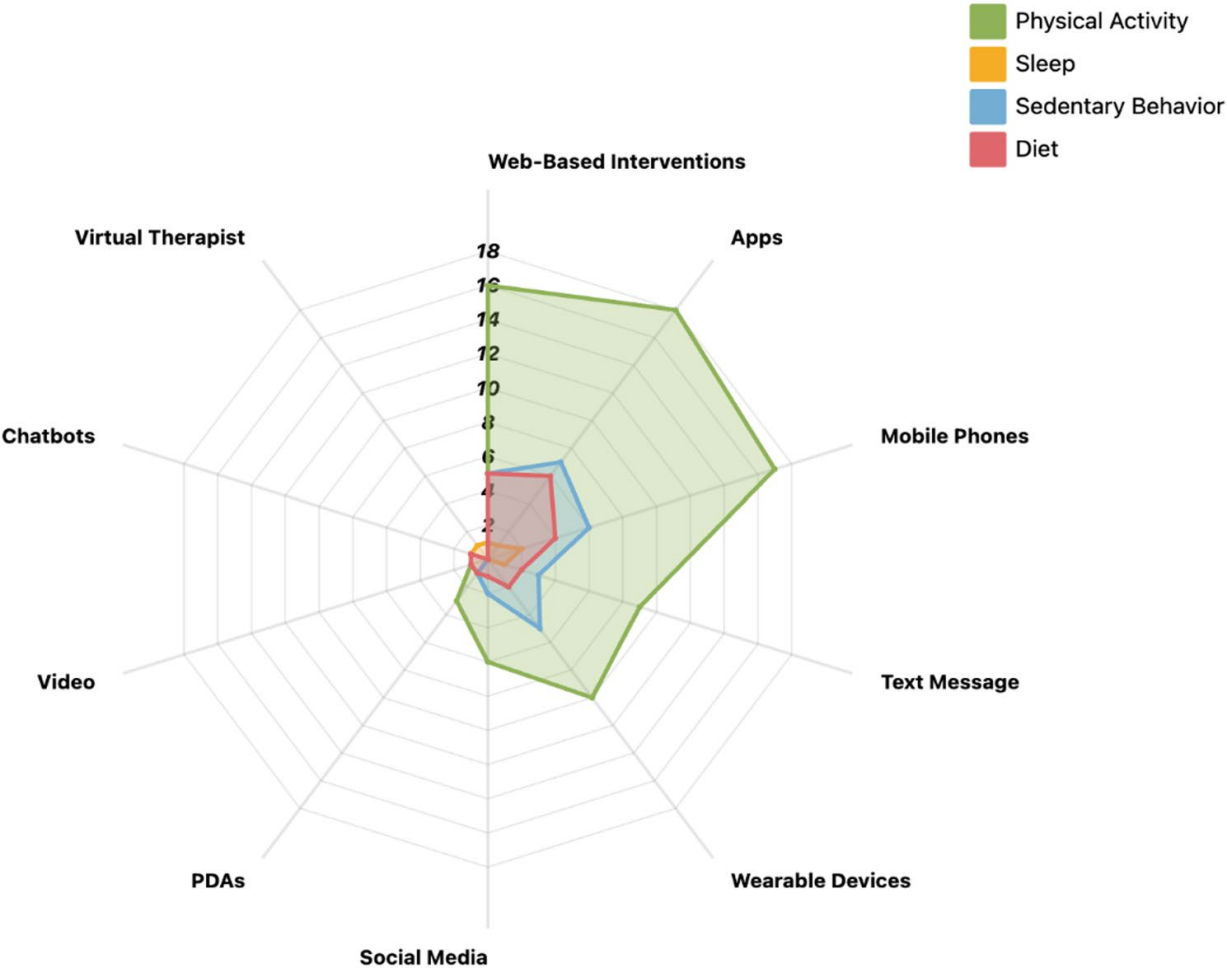
Target Behaviors and Digital Health Technologies

Different target behavioral domains demonstrate both convergence and divergence in the types of digital health intervention technologies employed (see Fig. 4). Physical activity interventions most extensively rely on mobile applications, wearable devices, and personalized feedback systems [13, 16, 56, 58–60, 62, 70]. These technologies typically require continuous internet connectivity, advanced digital literacy, and stable device conditions, which is associated with higher usage rates and greater intervention effectiveness among high- education and high-income populations [19, 57, 75, 82]. Conversely, low-resource populations experience more pronounced constraints related to device access, data affordability, and operational skills [19, 57, 61, 92]. In contrast, dietary interventions more frequently adopt lower-technology delivery modes, such as text messages, phone calls, and emails [56, 64, 80, 87, 89–91]. These intervention formats demonstrate comparatively higher accessibility among rural populations, older adults, and individuals with lower education [56, 88, 90]. Such approaches can sustain relatively stable participation even in settings characterized by limited internet access or insufficient digital literacy, thereby making them a preferred intervention strategy in resource-constrained contexts [97]. Digital health tools targeting sedentary behavior and sleep remain relatively limited, and are often centered on web-based interventions [71] or simple monitoring devices [19, 93]. Existing evidence suggests that these tools frequently lack personalized and diversified design features, as well as sufficient technical compatibility and cultural adaptability, thereby constraining intervention coverage and equality [19, 66, 71, 93].

### Reporting of equality related design and implementation features

Building upon the identification of differential outcomes among diverse social groups in digital health interventions, this study further examined whether the included reviews reported intervention design and implementation characteristics explicitly related to equality considerations. These characteristics encompassed aspects such as interface adaptation, content simplification, adjustment of usage burden, and the provision of support systems. Overall, reporting of such information was infrequent and lacked systematic consistency across the existing reviews. Among the 41 included reviews, only five explicitly reported design arrangements related to accessibility or usability when describing intervention effects or group differences [19, 42, 57, 61, 81]. These reports primarily focused on access-level support measures, such as device or network availability, or content adaptations targeting older adults. In contrast, reports addressing whether interventions were adapted in terms of task structure, interaction pacing, information density, or supportive mechanisms were extremely limited. Even when Table 2 demonstrated significant disadvantages for older adults, low-income, low-education, or rural populations in participation rates, adherence, and long-term benefits, these disparities were rarely explicitly attributed to specific intervention design or implementation characteristics. This pattern was observed consistently across different target behavior domains. Whether examining physical activity, dietary interventions, or sedentary behavior and sleep interventions, systematic review analyses predominantly focus on population-level disparities, while offering limited insight into whether and how interventions mitigate these disparities through design features and support mechanisms. Consequently, existing evidence more clearly documents real-world disparities in participation and intervention benefits, yet comprehensive evidence directly linking these disparities to specific intervention design and implementation features remains scarce.

## Discussion

### Social inequality indicators interpreted through the PROGRESS-Plus framework

This study employs the PROGRESS-Plus framework to systematically identify and categorize social inequality indicators in adult lifestyle behaviors facilitated by digital health interventions. As demonstrated in the Results section, the included reviews extensively documented disparities across diverse social groups in digital health interventions, encompassing multiple indicators such as age, gender, education, income, place of residence, and digital health literacy. For instance, older adults often encounter difficulties in operating digital devices [85, 98, 99], individuals with lower education or limited digital literacy are more likely to discontinue participation during early intervention stages [100], and those with limited income may experience reduced participation opportunities due to device and internet-related costs [10, 22]. These findings indicate that digital health interventions exhibit pronounced group-based disparities in real-world implementation contexts. However, the results also indicate that these disparities are primarily documented at the outcome level, rather than being examined through intervention processes. Most reviews do not report intervention design or implementation features explicitly related to equality, including interface adaptation, content simplification, or adjustments to usage burden. Therefore, while this study reveals which groups are more likely to be disadvantaged in digital health interventions, existing evidence remains insufficient to determine whether these differences arise from unequal participation conditions embedded within intervention design or implementation processes. Within these evidentiary boundaries, interpreting outcomes solely through observed group disparities risks reducing social inequality to individual-level deficiencies in capability, resources, or motivation, while overlooking potential structural limitations in intervention design and implementation. It is precisely in this context that existing research advocates a conceptual shift from asking “which groups perform worse” to examining “whether interventions provide comparable accessibility and reachability across different populations” [57, 92].

Within the broader literature on digital health and social inequality, existing research suggests that digital health interventions generally adopt three broad categories of strategies to address social inequality. The first category of strategies centers on compensating for gaps in access and affordability, including device lending or subsidy programs [10], data plan support [62], and community kiosks or offline intervention formats [63]. While these measures have been shown to reduce device- and connectivity-related disparities in controlled trial settings, research generally lacks rigorous evaluations of their effectiveness in real-world implementation contexts [18, 75, 101]. This pattern suggests that current access-support strategies more closely resemble intensive, trial-specific measures rather than equal solutions that are viable for long-term, routine implementation in real-world settings. The second category of strategies focuses on the adaptation of information presentation and intervention content. Research demonstrates that interface simplification tailored to age, cultural background, and literacy level [98], along with visualization strategies and multilingual support [69], as well as culturally sensitive content expression [102], can significantly enhance user experience among specific population groups. While such design approaches are effective in reducing cognitive load, existing adaptations primarily target older adults [81, 100, 103], whereas adaptations targeting users with lower education or unemployed populations remain scarce. Furthermore, most interventions remain limited to content-level adjustments, without addressing deeper design dimensions such as task structure, information density, or feedback pacing [102, 104]. The third category of strategies involves the provision of technical support and broader contextual arrangements. Some studies have facilitated sustained engagement among time-constrained or medically complex users through ongoing technical assistance [105], peer support groups [106], behavioral coaching follow-ups [107], or flexible task completion windows [81]. However, such measures often rely heavily on external human resources, which limits their scalability within routine healthcare and public health systems. It should be noted that these strategies are primarily derived from individual empirical studies or theoretical discussions, rather than being directly synthesized from the findings of the present review. In contrast to our findings, although some reviews sporadically mention device support, interface simplification, or technical assistance when describing intervention contexts [19, 82, 83], such information is inadequately reported at the review level, lacks standardized reporting practices, and is rarely leveraged to explain group-level outcome differences.

Further analysis indicates that current digital health interventions predominantly address the most readily identifiable forms of social inequality indicators, such as age-related operational difficulties or language comprehension needs [69, 98]. In contrast, indicators with more profound influences on health behaviors, such as income, employment, health condition, are most often operationalized solely as inclusion criteria or statistical control variables [57, 104], and rarely translated into actionable intervention design elements. This approach risks obscuring the ways in which complex life circumstances constrain user engagement, thereby perpetuating disadvantages among individuals facing multiple, intersecting adversities within interventions. For instance, many trials require smartphone ownership and basic digital literacy as prerequisites for participation—a design choice that implicitly excludes low-income individuals, those without reliable access to devices, or those experiencing unstable internet connectivity, thereby limiting the extent to which study samples reflect the diversity of real-world populations [10, 108]. Digital health interventions targeting individuals with multiple coexisting conditions often impose additional monitoring and self-management tasks without corresponding reductions in interface complexity or overall operational burden. Consequently, the groups most in need of intervention are subject to heightened usage demands and participation pressures [75, 101]. Similarly, existing interventions often lack flexibility in task pacing, feedback windows, and modes of interaction for users bearing substantial caregiving responsibilities or working irregular schedules. Such interventions generally presuppose that users can complete tasks at fixed or predefined times, potentially overlooking the impact of time constraints on sustained participation [81, 103]. Collectively, these issues reveal that despite claims of formal universality, digital health interventions may be fundamentally designed for resource-rich, time-flexible, and digitally proficient users. Under such circumstances, nominal openness does not necessarily translate into substantive accessibility across diverse social contexts, leaving the most vulnerable users facing greater barriers to intervention entry, operation, and sustained adherence [57, 109, 110]. This phenomenon aligns with the core argument advanced by Veinot et al. [40], which suggests that technologies lacking adaptive mechanisms may actively exacerbate existing social inequality. Therefore, understanding variations in the effectiveness of digital health interventions requires shifting attention away from users’ willingness or ability to use technology, toward examining whether interventions adequately anticipate and mitigate context-specific resistance factors during the design phase.

Therefore, a core finding of this study lies not in determining whether existing digital health interventions lack equal design, but in demonstrating that evidence at the review level primarily documents social inequality indicators at the outcome level, while rarely providing information on how interventions are designed and implemented to address these inequality indicators. In the absence of such information, assessments of intervention equality remain highly constrained. From a methodological perspective, this gap is unlikely to be incidental and likely stems from multiple contributing indicators. First, trial recruitment processes themselves exhibit substantial selection bias. Many randomized controlled trials exclude potential participants with limited access to devices, significant time constraints, or complex health conditions during the enrollment phase [108, 111, 112]. This practice may result in subsequent evidence disproportionately reflecting the experiences of better-resourced users, thereby failing to capture the diversity of real-world populations. Second, digital health tools commonly rely on existing technological infrastructures to develop functional modules. Interface organization and feedback mechanisms frequently follow design patterns optimized for so-called typical users, making it costly to redesign tasks or interaction flows for disadvantaged groups [112–114]. Third, many platforms implicitly assume that users possess stable devices, adequate digital literacy, and consistent schedules during the design phase [40]. These assumptions systematically exclude the needs of resource-constrained users or those in complex life situations from both design and evaluation processes [115]. Finally, critical determinants of behavior change, such as employment patterns and life stressors, are frequently reduced to statistical control variables, given the difficulty of translating them into standardized experimental parameters, and are rarely incorporated into intervention design itself [104, 116].

### Behavior-specific patterns and social inequality indicators

This study further examines differences across distinct target behavioral domains within digital health interventions. The findings indicate that digital health interventions are not randomly distributed across behavioral domains, but instead form relatively stable configurations shaped by behavioral characteristics, monitoring requirements, participation modalities, resource conditions, and prevailing research traditions. Understanding these behavior-specific configurations is essential for explaining observed disparities in equality across different behavioral domains.

First, in terms of evidence distribution, the field of physical activity is characterized by relatively comprehensive reporting on social inequality indicators, whereas the domains of diet, sedentary behavior, and sleep exhibit a marked lack of systematic discussion on related issues. Although studies on dietary interventions do address disparities related to income and education, they predominantly focus on adoption patterns among resource-rich populations [56, 81, 88–91], with limited attention given to the barriers faced by low-resource populations in accessing and sustaining such interventions. Evidence on equality in sedentary behavior and sleep interventions is even more limited, with existing reviews primarily examining technological efficacy or short-term outcomes [14, 19, 57, 60, 66], while largely lacking analysis of accessibility across different social groups. Given this substantial evidence gap, these behavioral domains cannot be directly compared with areas in which equality has been more extensively studied. Nevertheless, this absence of evidence raises concerns that certain vulnerable populations may be systematically marginalized without adequate recognition.

Second, the results reveal distinct patterns in the forms of intervention adopted across different behavioral domains. It is important to note that this study’s presentation of these patterns primarily focuses on differences in technological configurations, rather than on their underlying causes. The underlying causes of these patterns, as well as their mechanistic relationships with social inequality indicators, require further interpretation through engagement with the broader literature. Existing research suggests that technology selection may be shaped by multiple interrelated factors, including the following: (1) Behavioral characteristics and data requirements. Physical activity behaviors are highly quantifiable and generate immediate feedback, such as step count, heart rate, and activity duration, making them well suited for integration with digital health tools that require continuous monitoring and dynamic feedback [117, 118]. In contrast, dietary behaviors depend largely on subjective self-reporting and periodic reminders, and are therefore typically supported through lower-effort, information-delivery focused approaches [73, 80, 91]. Sleep and sedentary behavior monitoring are largely device-dependent, yet corresponding interventions may not require frequent user interaction, leading research to focus more on measurement itself rather than on complex behavioral shaping processes. This pattern indicates that technology selection is primarily determined by the observability of the target behavior and its feedback requirements. (2) User engagement methods. Different behavioral interventions demand varying levels of active user participation, depending on their intended mechanisms of behavior change. For instance, physical activity interventions typically require users to engage in tasks, record behaviors, or respond to feedback, and thus more frequently employ highly interactive technological tools. Dietary interventions, conversely, often rely on reminders and prompts, with relatively lower dependence on sustained active user input. This implies that technological configurations often reflect differences in the behavioral burden imposed on users by interventions, rather than differences in technological sophistication. (3) Resource conditions and population characteristics. Certain intervention formats are only suitable for populations with stable access to devices, strong digital skills, and high mobility, such as wearable monitoring devices [16, 60, 72, 76, 81, 83, 85, 87] and social or intelligent interactive tools [19, 75, 82]. In contrast, populations with limited resources are more likely to rely on simple, low-barrier interventions that do not disrupt daily routines, such as communication- and messaging-based approaches [56, 64, 80, 87, 89–91]. Thus, technological choices often entail differing entry barriers, and participation is determined not by the technology, but by whether different populations possess the resources and capacities required to overcome these barriers [119]. (4) Research traditions and path dependence. Physical activity research has long relied on monitoring tools such as pedometers and accelerometers, with its digital evolution naturally extending toward mobile applications and wearable devices [120]. Dietary research has traditionally emphasized reminders, interviews, and behavioral prompts, and therefore continues to rely on communication-based methods such as text messages and phone calls [73, 80, 91]. The historical development of these research paradigms has generated path dependencies in technology adoption across behavioral domains. (5) Privacy, ethics, and user burden considerations. In certain behavioral domains, although more sophisticated technological tools may be theoretically feasible, their implementation often involves privacy-sensitive data, perceptions of intrusive monitoring, or high learning costs [42, 84], thereby hindering widespread adoption. For instance, sleep-related data constitute highly sensitive personal information, and frequent behavioral recording may increase user burden or intensify privacy concerns. Collectively, these factors further shape the adoption and acceptability of intervention formats across different behavioral domains.

### Structural mechanisms of digital exclusion: a critical perspective

The findings of this study indicate that systematic disparities persist in intervention benefits among different social groups in digital health interventions. Furthermore, most reviews do not report intervention design and implementation characteristics explicitly related to equality. Within this evidentiary gap, this study introduces the analytical perspective of digital exclusion to provide an explanatory framework for why disadvantaged groups, despite having nominal or formal access, consistently lag behind in real-world intervention engagement. It should be noted that this section constitutes an interpretive comparison and theoretical extension grounded in the study’s findings, intended to propose potential mechanisms rather than to conflate theoretical inferences with direct empirical conclusions drawn from this research.

### Theoretical foundation: from the digital divide to digital exclusion

Van Dijk’s [119] digital divide theory posits that inequality arising from digital technologies manifest along three core dimensions: access, capability, and meaningfulness of use. However, recent research further emphasizes that inequality does not stem solely from users’ lack of skills or resources, but often originates from the assumptions embedded in technological systems during the design phase [104, 113, 114, 116]. In other words, inequality may be embedded within technological systems prior to actual use. Many digital health platforms rely on an “ideal user model” during development, assuming that users possess proficiency with digital devices, stable internet and terminal access, sufficient time and motivation to engage with interventions, and the capacity to comprehend medical, nutritional, or exercise science terminology used on the platform [40, 121]. These assumptions often reflect the experiences and expectations of developers, healthcare professionals, or high-education groups, rather than those of the broader or more heterogeneous user population. This “ideal user model” has at least two significant consequences. First, it overlooks the constraints faced by disadvantaged groups in terms of device access, operational experience, and cognitive load, thereby placing them at a disadvantage from the outset [113, 114]. Second, it leads platforms to privilege more capable users in process design and content presentation, thereby inadvertently widening disparities between social groups. Even when platforms claim universal accessibility, they may generate de facto usage biases, leaving certain groups “left behind” at various stages of access, operation, or comprehension [122–124]. Thus, the shift from the digital divide to digital exclusion is not merely a conceptual transition, but rather a reorientation of analytical focus from individual user characteristics toward how technological systems construct rule frameworks governing “who can use, who can use more easily, and who can ultimately benefit from use.” It is precisely in this sense that the concept of digital exclusion provides a more analytically powerful perspective for explaining persistent social inequality indicators.

### The triple mechanism of digital exclusion

Building upon the aforementioned theoretical perspectives, this study identifies three interrelated mechanisms underlying digital exclusion in digital health interventions. These mechanisms do not stem from users’ inability or unwillingness to engage with technology, but rather from barriers arising at the intersection of technical design, task requirements, and users’ lived experiences. Ultimately, these barriers produce differentiated user experiences and intervention outcomes for different social groups operating within the same platform. (1) Access barriers. Access barriers encompass constraints such as inadequate devices, high data costs, and poor network coverage, representing the most fundamental yet critical hurdles. For low-income households, rural residents, or older adults, the ability to access the platform often determines whether participation in digital health interventions is possible at all. For instance, underpowered smartphones, limited video capabilities, or prohibitively expensive data plans may prevent users from accessing digital health tools at the initial stage. When access is constrained, subsequent use and potential benefits become largely unattainable. (2) Capability barriers. Even after gaining access, many users encounter substantial difficulties during operation and interaction. Complex interfaces, abstract task flows, and content laden with technical jargon often demand higher levels of literacy, digital experience, and health-related knowledge. Research indicates that platform language styles and information presentation tend to be oriented toward highly educated users [100]. As a result, older adults, individuals with lower education, or those lacking digital health literacy are more likely to experience repeated operational breakdowns, struggle to sustain engagement, and gradually reduce their willingness to continue using the platform. (3) Meaningfulness barriers. The third category of barriers emerges when users are technically able to access and operate the platform. Research indicates that even when vulnerable groups successfully complete required operations, they may still derive limited practical value from the platform [124, 125]. This limitation stems from platform recommendations, goals, and feedback frequently failing to align with users’ real-life contexts, such as through impractical suggestions, excessive action costs, or limited cultural and lifestyle relevance. Consequently, users may complete prescribed usage processes yet lack sustained motivation, thereby limiting the likelihood of achieving meaningful and lasting behavioral change.

### Conceptual model

**Fig. 5:**
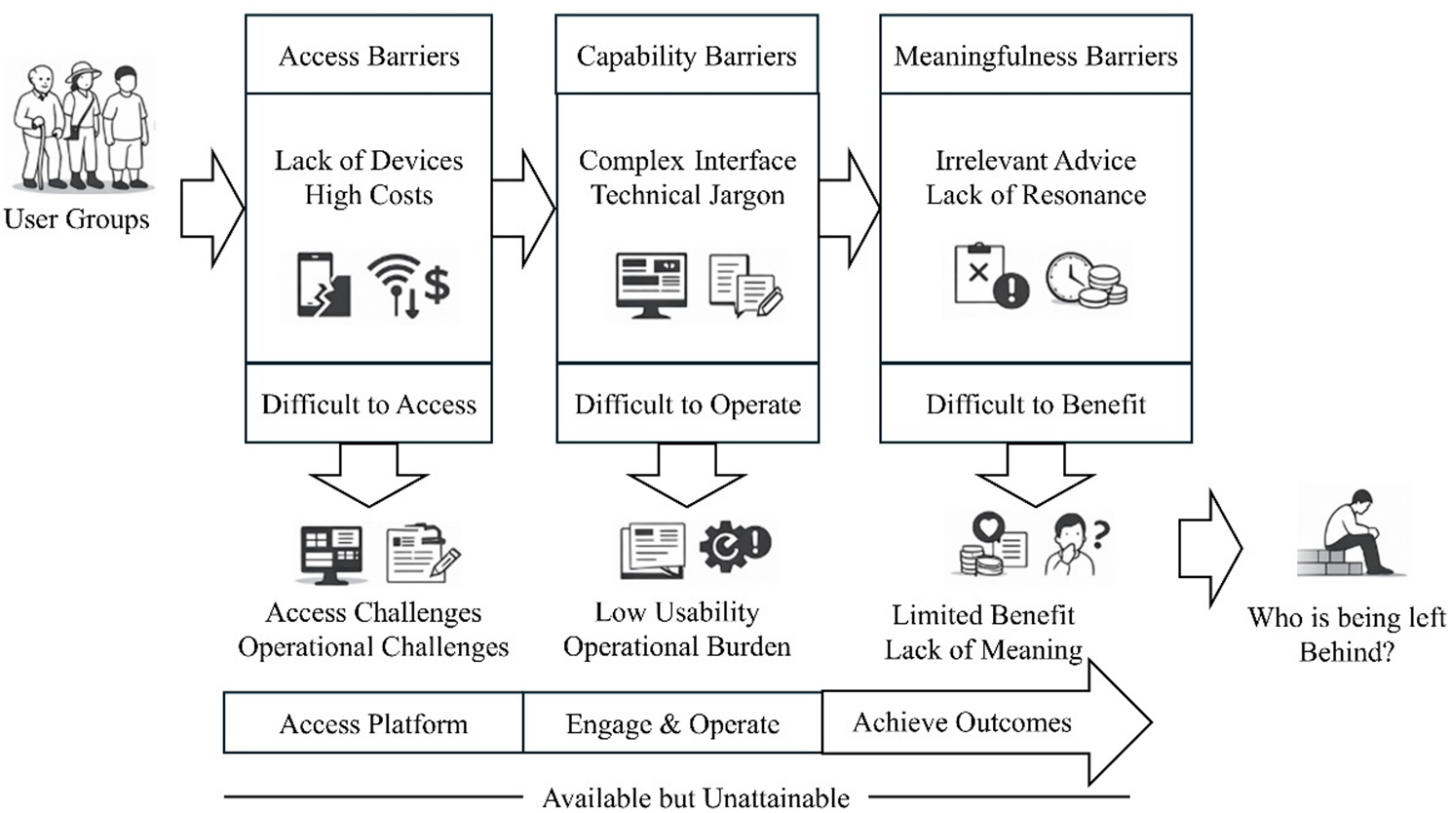
The Conceptual Model of Digital Exclusion

The three mechanisms described above jointly contribute to the phenomenon often described as “available but unattainable” in digital health interventions: while platforms are formally open to all users, many continue to struggle to obtain meaningful support during actual use. In other words, the problem lies not solely in final intervention outcomes, but often originates from unequal access and usage conditions embedded within platform design. Based on this understanding, this study proposes a conceptual model of digital exclusion (see Fig. 5) to illustrate how digital health interventions progressively accumulate social inequality across successive stages of use. This model highlights two core issues. First, usage disparities do not stem from users’ “inadequate capabilities,” but rather from platform operational mechanisms that introduce implicit barriers across access, operation, and comprehension, thereby rendering certain groups more vulnerable to falling behind. Second, digital health technologies are not inherently neutral; depending on design choices, they may either exacerbate existing disparities or facilitate higher levels of inclusivity. The ways in which platforms structure processes, present information, and design tasks directly shape whether interventions can achieve substantive equality. Thus, this model not only accounts for the formation of digital exclusion, but also provides a directional analytical framework for evaluating and improving digital health interventions. To advance toward substantive equality, platforms must simultaneously ensure simplicity and clarity across the three critical stages of “access-capability-meaningfulness,” thereby enabling users with diverse abilities and backgrounds to benefit from digital health interventions.

### Evidence gaps and theoretical omissions

Based on the foregoing analysis, existing review studies addressing social inequality indicators in digital health interventions continue to exhibit substantial evidence gaps. First, the majority of reviews fail to conceptualize social inequality as a core analytical variable; instead, indicators such as age, gender, and SES are typically reported only passively within heterogeneity or subgroup analyses. Second, despite the well-established status of education, income, and SES as critical social determinants of health behavior [26, 126], these indicators have received insufficient analytical attention in the majority of reviews. In some cases, these indicators are reduced to mere background descriptors or treated solely as control variables, thereby neglecting their pivotal influence on intervention accessibility, utilization capacity, and the generation of health outcomes. Third, with respect to the temporal dimension of digital health interventions, current research predominantly concentrates on short-term effectiveness assessments, with limited tracking and evaluation of dynamic variations in intervention sustainability, long-term adherence, and enduring health benefits across diverse populations. This limitation constrains the capacity to ascertain whether digital health interventions exacerbate, mitigate, or transform existing social inequality over extended periods, thereby reducing the foresight available to policy-making processes.

### Strengths and limitations

This study represents one of the first systematic umbrella reviews to explicitly integrate digital health interventions with a social inequality perspective, offering several methodological and conceptual strengths. First, this study explicitly adopts social inequality indicators as its core analytical lens and employs the PROGRESS-Plus framework as a structured data extraction tool. This approach enables a comprehensive identification and synthesis of inequality-related indicators that were previously discussed in a fragmented manner or not systematically addressed in existing reviews. While this study cannot fundamentally alter the historical marginalization of social inequality indicators within prior literature, its systematic integration of evidence across diverse reviews contributes to increasing the visibility and analytical salience of this issue within the field of digital health intervention research. Second, building upon evidence synthesized from systematic reviews, this study maps the relationships between different types of digital health interventions and their corresponding target behaviors. By integrating digital divide theory, this study proposes a conceptual model of three interrelated barriers “access-capability-meaningfulness” to explain the critical stages at which digital exclusion may emerge. While the umbrella review design precludes direct causal testing of these mechanisms, it provides a conceptually grounded analytical framework for future research. This framework can guide subsequent empirical studies to systematically examine how access, capability, and contextual fit shape intervention experiences and outcomes across diverse populations during intervention design, measurement, and evaluation.

However, this study is subject to several limitations. First, the study population primarily comprised adults, thereby excluding children and adolescents. Given the substantial differences across age groups in lifestyle behaviors, digital health tool usage patterns, and the mechanisms underlying the formation of social inequality indicators, this demographic restriction partially limits insights into the life-course characteristics of social inequality. It also constrains the extent to which the study’s conclusions can fully capture age-specific manifestations of inequality in digital health interventions. Second, this study applied stringent inclusion and exclusion criteria, incorporating only English-language systematic reviews and comprehensive evidence, while excluding grey literature and highly contextualized small-sample studies. While this approach enhanced quality control and comparability, it may have led to the omission of relevant evidence related to social inequality, thereby limiting the overall comprehensiveness of the findings. Third, the included reviews exhibited substantial heterogeneity in methodological quality. Based on AMSTAR-2 assessments, only two reviews were rated as having high methodological confidence. Methodological shortcomings in some reviews may compromise the robustness of the synthesized conclusions. Fourth, because existing reviews largely failed to systematically incorporate social inequality as an analytical dimension, this study faced limitations in data extraction and categorization. Consequently, certain indicator-level analyses may have underestimated or incompletely captured relevant inequality indicators.

### Implications for practice and policy

First, at the level of intervention practice, the design and implementation of digital health interventions require a conceptual shift from “usability” to “accessibility.” Our findings indicate that older adults, individuals with lower education and income, rural residents, and those with lower digital health literacy consistently exhibit lower participation rates and adherence across multiple behavioral domains. Yet these disparities are rarely explicitly linked to specific intervention design or implementation characteristics. This suggests that practitioners should not evaluate digital health tools solely on the basis of technical completeness or interface friendliness when developing or adopting them. Instead, practitioners should systematically assess the entry barriers and operational burdens that interventions impose on different population groups under real-world usage conditions. Second, practitioners must guard against the tendency to attribute participation disparities primarily to individual-level motivation or capability deficits. Numerous reviews document low engagement and poor adherence among vulnerable populations, yet seldom report whether interventions were adapted in terms of task pacing, feedback frequency, or support modalities. This implies that, without critically examining the demands imposed by interventions themselves, practitioners may inadvertently shift responsibility onto individual users, thereby undermining the inclusivity of digital health interventions. Third, policymakers should prioritize equality considerations within the design and evaluation frameworks of digital health interventions. Existing reviews demonstrate extremely limited and inconsistent reporting on equality-related design features, such as interface adaptation, content simplification, and usage burden adjustment, with no unified reporting standards.This suggests that policymakers could consider explicitly requiring reporting on accessibility arrangements for different social groups, rather than focusing solely on overall intervention effectiveness, when funding, approving, or promoting digital health projects. Such an approach would reposition equality from a secondary consideration to a core evaluation metric within digital health intervention governance.

### Implications for future research

Current research on social inequality indicators in digital health interventions remains at an early stage, with substantial scope for future expansion across several directions. First, most studies treat differences in age, income, or education primarily as statistical control variables, rather than adopting experimental designs that position social inequality as a core research question. Future studies should prioritize social inequality as a focal analytic dimension within prospective registries, intervention pathway analyses, and randomized trial designs, systematically examining group-based disparities in access, operational burden, interaction modalities, and behavioral change processes. Second, existing research often focuses on single-indicator forms of social inequality, whereas real-world disadvantage typically arises from multiple intersecting indicators, such as low income, female gender, rural background, and health conditions, that collectively shape digital health intervention pathways. Future research should employ interaction-effect modeling and mixed-methods designs to identify barriers encountered by multiply vulnerable groups at critical intervention junctures and to elucidate the mechanisms underlying these barriers. Third, current research predominantly relies on cross-sectional descriptions, with limited longitudinal tracking and insufficient attention to mechanism construction. Future studies should integrate digital health intervention research with social mobility research, structural theories of health inequality, and models of technology adoption processes. Such integration would enable analyses of the dynamic relationships between income, education, employment, and health behavior transformation, while also elucidating the mechanisms operating within intervention pathways. Fourth, the existing literature predominantly originates from Western countries, resulting in an imbalance within the global knowledge system on digital health equality. In resource-constrained countries, disparities in infrastructure, cultural contexts, and policy environments may give rise to distinct equality challenges. Future research should conduct empirical studies in low- and middle-income countries to strengthen global comparative perspectives and to provide a more diverse evidence base for international digital health governance.

## Conclusions

As digital health interventions continue to proliferate globally, they have exhibited considerable potential for fostering healthy lifestyle behaviors. However, the present study identifies that the prevailing design of digital health interventions remains inadequate in achieving inclusivity, as certain populations are marginalized due to technical barriers, economic constraints, and cultural incongruities, thereby contributing to the emergence of a “left-behind” population. Such social inequality indicators not only diminish the effectiveness of digital health interventions but may also inadvertently exacerbate pre-existing health disparities, thereby engendering novel forms of social exclusion embedded within digital health technologies. Therefore, future digital health interventions should urgently adopt a equality-focused paradigm across multiple indicators, encompassing design philosophy, resource allocation, policy frameworks, and evaluation mechanisms. While maintaining an emphasis on technological efficacy, it is imperative to concurrently acknowledge and address the heterogeneous needs of diverse social groups, thereby facilitating the development of more inclusive, targeted, and sustainable health promotion frameworks.

## Supplementary Information

Supplementary Material 1.

Supplementary Material 2.

Supplementary Material 3.

Supplementary Material 4.

Supplementary Material 5.

Supplementary Material 6.

Supplementary Material 7.

Supplementary Material 8.

## Data Availability

Data sharing is not applicable to this article as no datasets were generated or analysed during the current study.

## Abbreviations

WHO: World Health Organization
OECD: Organisation for Economic Co-operation and Development
N: Number of participants
SES: Socioeconomic Status
NR: Not Reported
PDAs: Personal Digital Assistants
DVDs: Digital Videodisks
SMS: Short Message Service
