# Supplementary material for: Digital health interventions for promoting adults lifestyle behaviors: who is being left behind? An evidence synthesis of social inequality
Source: Int J Behav Nutr Phys Act. 2026 Feb 6;23:23. doi: 10.1186/s12966-026-01874-4 (PMC12977709; doi:10.1186/s12966-026-01874-4)
Supplement: Supplementary file 1 — Supplementary Material 1. [file 12966_2026_1874_MOESM1_ESM.docx]

| **Database** |  | **Query** |
| --- | --- | --- |
| Pub Med |  | ( "web-based"[Title/Abstract] OR "internet-based"[Title/Abstract] OR "technology-based"[Title/Abstract] OR  "ehealth"[Title/Abstract] OR "mhealth"[Title/Abstract] OR "digital health"[Title/Abstract] OR  "digital intervention"[Title/Abstract] OR "telehealth"[Title/Abstract] OR "telemedicine"[Title/Abstract] OR  "connected health"[Title/Abstract] OR "mobile applications"[Title/Abstract] OR "mobile apps"[Title/Abstract] OR  "health app"[Title/Abstract] OR "wearable device"[Title/Abstract] OR "wearable technology"[Title/Abstract] OR  "activity tracker"[Title/Abstract] OR "SMS intervention"[Title/Abstract] OR "text message"[Title/Abstract] OR  "mobile phone–based"[Title/Abstract] OR "remote monitoring"[Title/Abstract] OR  "online intervention"[Title/Abstract] OR "social media"[Title/Abstract] OR  "online social networking"[Title/Abstract] OR "social media health"[Title/Abstract] OR  "personal digital assistant"[Title/Abstract] )  AND  ( "physical activity"[Title/Abstract] OR "exercise"[Title/Abstract] OR "active living"[Title/Abstract] OR  "movement"[Title/Abstract] OR "sedentary behavior"[Title/Abstract] OR "sitting time"[Title/Abstract] OR  "screen time"[Title/Abstract] OR "sleep"[Title/Abstract] OR "sleep duration"[Title/Abstract] OR  "sleep quality"[Title/Abstract] OR "healthy eating"[Title/Abstract] OR "diet"[Title/Abstract] OR  "nutrition"[Title/Abstract] OR "nutritional intake"[Title/Abstract] OR "fruit consumption"[Title/Abstract] OR  "vegetable intake"[Title/Abstract] OR "sugar consumption"[Title/Abstract] OR "caloric intake"[Title/Abstract] OR  "healthy diet"[Title/Abstract] OR "balanced diet"[Title/Abstract] OR "eating behavior"[Title/Abstract] OR  "eating pattern"[Title/Abstract] OR "food choice"[Title/Abstract] OR "lifestyle behavior"[Title/Abstract] OR  "behavior change"[Title/Abstract] OR "health behavior"[Title/Abstract] )  AND  ( "equit*"[Title/Abstract] OR "inequit*"[Title/Abstract] OR "inequalit*"[Title/Abstract] OR  "disparit*"[Title/Abstract] OR "equality"[Title/Abstract] OR "deprivation"[Title/Abstract] OR  "digital divide"[Title/Abstract] OR "digital exclusion"[Title/Abstract] OR  "technological exclusion"[Title/Abstract] OR "socioeconomic status"[Title/Abstract] OR "SES"[Title/Abstract] OR  "low income"[Title/Abstract] OR "rural population"[Title/Abstract] OR "older adults"[Title/Abstract] OR  "vulnerable population"[Title/Abstract] OR "at-risk population"[Title/Abstract] OR  "ethnic minorit*"[Title/Abstract] OR "social determinant"[Title/Abstract] OR "social gradient"[Title/Abstract] OR  "social stratification"[Title/Abstract] OR "structural inequity"[Title/Abstract] OR  "systemic inequity"[Title/Abstract] OR "marginalization"[Title/Abstract] OR  "access barrier"[Title/Abstract] OR "technology access"[Title/Abstract] )  AND  ( "systematic review"[Title/Abstract] OR "meta-analysis"[Title/Abstract] OR "scoping review"[Title/Abstract] OR  "umbrella review"[Title/Abstract] OR "narrative review"[Title/Abstract] OR "integrative review"[Title/Abstract] OR  "review of reviews"[Title/Abstract] OR "meta-synthesis"[Title/Abstract] ) |
| Web of Sience |  | TS=( ("web-based" OR "internet-based" OR "technology-based" OR "ehealth" OR "mhealth" OR "digital health" OR "digital intervention" OR  "telehealth" OR "telemedicine" OR "connected health" OR "mobile applications" OR "mobile apps" OR "health app" OR  "wearable device" OR "wearable technology" OR "activity tracker" OR "SMS intervention" OR "text message" OR  "mobile phone–based" OR "remote monitoring" OR "online intervention" OR "social media" OR "online social networking" OR  "social media health" OR "personal digital assistant")  AND  ("physical activity" OR "exercise" OR "active living" OR "movement" OR  "sedentary behavior" OR "sitting time" OR "screen time" OR  "sleep" OR "sleep duration" OR "sleep quality" OR  "healthy eating" OR "diet" OR "nutrition" OR "nutritional intake" OR  "fruit consumption" OR "vegetable intake" OR "sugar consumption" OR  "caloric intake" OR "healthy diet" OR "balanced diet" OR  "eating behavior" OR "eating pattern" OR "food choice" OR  "lifestyle behavior" OR "behavior change" OR "health behavior")  AND  ( "equit" OR "inequit" OR "inequalit" OR  "disparit" OR "equality" OR "deprivation" OR  "digital divide" OR "digital exclusion" OR  "technological exclusion" OR "socioeconomic status" OR "SES" OR  "low income" OR "rural population" OR "older adults" OR  "vulnerable population" OR "at-risk population" OR  "ethnic minorit" OR "social determinant" OR "social gradient" OR  "social stratification" OR "structural inequity" OR  "systemic inequity" OR "marginalization" OR  "access barrier" OR "technology access" )  AND  ("systematic review" OR "meta-analysis" OR "scoping review" OR "umbrella review" OR  "narrative review" OR "integrative review" OR "review of reviews" OR "meta-synthesis")) |
| SPORTDiscus | #1 | “web-based” OR “internet-based” OR “technology-based” OR “ehealth” OR “mhealth” OR “digital health” OR “digital intervention” OR “telehealth” OR “telemedicine” OR “connected health” OR “mobile applications” OR “mobile apps” OR “health app” OR “wearable device” OR “wearable technology” OR “activity tracker” OR “SMS intervention” OR “text message” OR “mobile phone–based” OR “remote monitoring” OR “online intervention” OR “social media” OR “online social networking” OR “social media health” OR “personal digital assistant” |
|  | #2 | “physical activity” OR “exercise” OR “active living” OR “movement” OR “sedentary behavior” OR “sitting time” OR “screen time” OR “sleep” OR “sleep duration” OR “sleep quality” OR “healthy eating” OR “diet” OR “nutrition” OR “nutritional intake” OR “fruit consumption” OR “vegetable intake” OR “sugar consumption” OR “caloric intake” OR “healthy diet” OR “balanced diet” OR “eating behavior” OR “eating pattern” OR “food choice” OR “lifestyle behavior” OR “behavior change” OR “health behavior” |
|  | #3 | “equit” OR “inequit” OR “inequalit” OR “disparit” OR “equality” OR “deprivation” OR “digital divide” OR "digital exclusion" OR "technological exclusion" OR "socioeconomic status" OR SES OR "low income" OR "rural population" OR "older adults" OR "vulnerable population" OR "at-risk population" OR "ethnic minorit" OR "social determinant" OR "social gradient" OR "social stratification" OR "structural inequity" OR "systemic inequity" OR "marginalization" OR "access barrier" OR "technology access" |
|  | #4 | "systematic review" OR "meta-analysis" OR "scoping review" OR "umbrella review" OR  "narrative review" OR "integrative review" OR "review of reviews" OR "meta-synthesis" |
|  | #5 | #1 AND #2 AND #3 AND#4 |
| Scopus |  | (TITLE-ABS-KEY("web-based" OR "internet-based" OR "technology-based" OR  "ehealth" OR "mhealth" OR "digital health" OR "digital intervention" OR  "telehealth" OR "telemedicine" OR "connected health" OR  "mobile applications" OR "mobile apps" OR "health app" OR  "wearable device" OR "wearable technology" OR "activity tracker" OR  "SMS intervention" OR "text message" OR "mobile phone–based" OR  "remote monitoring" OR "online intervention" OR "social media" OR  "online social networking" OR "social media health" OR  "personal digital assistant"))  AND  (TITLE-ABS-KEY("physical activity" OR "exercise" OR "active living" OR "movement" OR  "sedentary behavior" OR "sitting time" OR "screen time" OR  "sleep" OR "sleep duration" OR "sleep quality" OR  "healthy eating" OR "diet" OR "nutrition" OR "nutritional intake" OR  "fruit consumption" OR "vegetable intake" OR "sugar consumption" OR  "caloric intake" OR "healthy diet" OR "balanced diet" OR  "eating behavior" OR "eating pattern" OR "food choice" OR  "lifestyle behavior" OR "behavior change" OR "health behavior"))  AND  (TITLE-ABS-KEY("equit*" OR "inequit*" OR "inequalit*" OR "disparit*" OR  "equality" OR "deprivation" OR "digital divide" OR "digital exclusion" OR  "technological exclusion" OR "socioeconomic status" OR "SES" OR  "low income" OR "rural population" OR "older adults" OR  "vulnerable population" OR "at-risk population" OR "ethnic minorit*" OR  "social determinant" OR "social gradient" OR "social stratification" OR  "structural inequity" OR "systemic inequity" OR "marginalization" OR  "access barrier" OR "technology access"))  AND  (TITLE-ABS-KEY("systematic review" OR "meta-analysis" OR "scoping review" OR  "umbrella review" OR "narrative review" OR "integrative review" OR  "review of reviews" OR "meta-synthesis")) |
| Cochrane Library |  | ("web-based" OR "internet-based" OR "technology-based" OR  "ehealth" OR "mhealth" OR "digital health" OR "digital intervention" OR  "telehealth" OR "telemedicine" OR "connected health" OR  "mobile applications" OR "mobile apps" OR "health app" OR  "wearable device" OR "wearable technology" OR "activity tracker" OR  "SMS intervention" OR "text message" OR "mobile phone–based" OR  "remote monitoring" OR "online intervention" OR "social media" OR  "online social networking" OR "social media health" OR  "personal digital assistant"):ti,ab,kw  AND  ("physical activity" OR "exercise" OR "active living" OR "movement" OR  "sedentary behavior" OR "sitting time" OR "screen time" OR  "sleep" OR "sleep duration" OR "sleep quality" OR  "healthy eating" OR "diet" OR "nutrition" OR "nutritional intake" OR  "fruit consumption" OR "vegetable intake" OR "sugar consumption" OR  "caloric intake" OR "healthy diet" OR "balanced diet" OR  "eating behavior" OR "eating pattern" OR "food choice" OR  "lifestyle behavior" OR "behavior change" OR "health behavior"):ti,ab,kw  AND  ("equit*" OR "inequit*" OR "inequalit*" OR "disparit*" OR  "equality" OR "deprivation" OR "digital divide" OR "digital exclusion" OR  "technological exclusion" OR "socioeconomic status" OR "SES" OR  "low income" OR "rural population" OR "older adults" OR  "vulnerable population" OR "at-risk population" OR "ethnic minorit*" OR  "social determinant" OR "social gradient" OR "social stratification" OR  "structural inequity" OR "systemic inequity" OR "marginalization" OR  "access barrier" OR "technology access"):ti,ab,kw  AND  ("systematic review" OR "meta-analysis" OR "scoping review" OR  "umbrella review" OR "narrative review" OR "integrative review" OR  "review of reviews" OR "meta-synthesis"):ti,ab,kw |
| Google  Scholar |  | ("web-based" OR "internet-based" OR "technology-based" OR  "ehealth" OR "mhealth" OR "digital health" OR "digital intervention" OR  "telehealth" OR "telemedicine" OR "connected health" OR  "mobile applications" OR "mobile apps" OR "health app" OR  "wearable device" OR "wearable technology" OR "activity tracker" OR  "SMS intervention" OR "text message" OR "mobile phone–based" OR  "remote monitoring" OR "online intervention" OR "social media" OR  "online social networking" OR "social media health" OR  "personal digital assistant"):ti,ab,kw  AND  ("physical activity" OR "exercise" OR "active living" OR "movement" OR  "sedentary behavior" OR "sitting time" OR "screen time" OR  "sleep" OR "sleep duration" OR "sleep quality" OR  "healthy eating" OR "diet" OR "nutrition" OR "nutritional intake" OR  "fruit consumption" OR "vegetable intake" OR "sugar consumption" OR  "caloric intake" OR "healthy diet" OR "balanced diet" OR  "eating behavior" OR "eating pattern" OR "food choice" OR  "lifestyle behavior" OR "behavior change" OR "health behavior"):ti,ab,kw  AND  ("equit*" OR "inequit*" OR "inequalit*" OR "disparit*" OR  "equality" OR "deprivation" OR "digital divide" OR "digital exclusion" OR  "technological exclusion" OR "socioeconomic status" OR "SES" OR  "low income" OR "rural population" OR "older adults" OR  "vulnerable population" OR "at-risk population" OR "ethnic minorit*" OR  "social determinant" OR "social gradient" OR "social stratification" OR  "structural inequity" OR "systemic inequity" OR "marginalization" OR  "access barrier" OR "technology access"):ti,ab,kw  AND  ("systematic review" OR "meta-analysis" OR "scoping review" OR  "umbrella review" OR "narrative review" OR "integrative review" OR  "review of reviews" OR "meta-synthesis"):ti,ab,kw |
| ProQuest | #1 | “web-based” OR “internet-based” OR “technology-based” OR “ehealth” OR “mhealth” OR “digital health” OR “digital intervention” OR “telehealth” OR “telemedicine” OR “connected health” OR “mobile applications” OR “mobile apps” OR “health app” OR “wearable device” OR “wearable technology” OR “activity tracker” OR “SMS intervention” OR “text message” OR “mobile phone–based” OR “remote monitoring” OR “online intervention” OR “social media” OR “online social networking” OR “social media health” OR “personal digital assistant” |
|  | #2 | “physical activity” OR “exercise” OR “active living” OR “movement” OR “sedentary behavior” OR “sitting time” OR “screen time” OR “sleep” OR “sleep duration” OR “sleep quality” OR “healthy eating” OR “diet” OR “nutrition” OR “nutritional intake” OR “fruit consumption” OR “vegetable intake” OR “sugar consumption” OR “caloric intake” OR “healthy diet” OR “balanced diet” OR “eating behavior” OR “eating pattern” OR “food choice” OR “lifestyle behavior” OR “behavior change” OR “health behavior” |
|  | #3 | “equit” OR “inequit” OR “inequalit” OR “disparit” OR “equality” OR “deprivation” OR “digital divide” OR "digital exclusion" OR "technological exclusion" OR "socioeconomic status" OR SES OR "low income" OR "rural population" OR "older adults" OR "vulnerable population" OR "at-risk population" OR "ethnic minorit" OR "social determinant" OR "social gradient" OR "social stratification" OR "structural inequity" OR "systemic inequity" OR "marginalization" OR "access barrier" OR "technology access" |
|  | #4 | "systematic review" OR "meta-analysis" OR "scoping review" OR "umbrella review" OR  "narrative review" OR "integrative review" OR "review of reviews" OR "meta-synthesis" |
|  | #5 | #1 AND #2 AND #3 AND#4 |
