# Supplementary material for: Digital health interventions for promoting adults lifestyle behaviors: who is being left behind? An evidence synthesis of social inequality
Source: Int J Behav Nutr Phys Act. 2026 Feb 6;23:23. doi: 10.1186/s12966-026-01874-4 (PMC12977709; doi:10.1186/s12966-026-01874-4)
Supplement: Supplementary file 5 — Supplementary Material 5. [file 12966_2026_1874_MOESM5_ESM.docx]

**Identification of studies via databases and registers**

**Identification of studies via other methods**

Records removed *before screening*:

Duplicate records removed

(n = 1531)

Records removed for other reasons (n = 0)

Records identified from*:

Databases (n = 3846)

- Web of Science (n= 530)
- PubMed (n = 260)
- Scopus (n = 672)
- Google Scholar (n = 871)
- SPORTDiscus (n = 532)
- ProQuest (n = 974)
- Cochrane Library (n = 7)

Records identified from:

Citation searching (n = 28)

**Identification**

Records excluded

(n = 1770)

Records screened

(n = 2315)

Reports not retrieved

(n = 43)

Reports not retrieved

(n = 3)

Reports sought for retrieval

(n = 28)

Reports sought for retrieval

(n = 545)

**Screening**

Reports excluded:

Did not report lifestyle behavior outcomes (n = 203)

Wrong target population

(n = 97)

Did not report social inequities

(n = 88)

Study population or setting too narrowly defined (n = 53)

Publication type not eligible

(n = 29)

Reports assessed for eligibility

(n = 25)

Reports excluded:

Did not meet inclusion criteria (n = 11)

Wrong population (n = 4)

Protocol/editorial/non-peer-reviewed (n = 1)

Reports assessed for eligibility

(n = 502)

Studies included in review

(n = 41)

**Included**
